# Complex evolution of East Asian Tertiary relict species revealed by the phylogeography of *Lindera obtusiloba*

**DOI:** 10.1186/s12870-025-07827-6

**Published:** 2025-12-09

**Authors:** Jun-Wei Ye, Rui Yang, Lei Bao, Meng-Jing Dai, Hong-Fang Wang, Jian-Ping Ge

**Affiliations:** 1 Key Laboratory of Forest Resources Conservation and Utilization in the Southwest Mountains of China, Ministry of Education/Key Laboratory for Conserving Wildlife with Small Populations in Yunnan/College of Forestry, Southwest Forestry University, Kunming, 650224 China; 2 National Forestry and Grassland Administration Key Laboratory for Conservation Ecology of Northeast Tiger and Leopard, Ministry of Education Key Laboratory for Biodiversity Science and Engineering & College of Life Sciences, Beijing Normal University, Beijing, 100875 China

**Keywords:** East Asia, Ecological adaptation, Diversification, *Lindera obtusiloba*, Refugia, Tertiary relicts

## Abstract

**Background:**

In East Asia, the evolutionary histories of Tertiary relicts are complex, involving not only southward retreats during the middle and late Tertiary but also repeated migrations northward or southward during the Quaternary. Three possible scenarios are hypothesized: High-Latitude Origin, Low-Latitude Origin, and Vicariance Origin. The phylogeography of *Lindera obtusiloba* is investigated using 27 independent nuclear low-copy genes to test these scenarios and explore key diversification processes.

**Results:**

The genetic diversity and effective population size at low latitudes were approximately two/three times greater than those at high latitudes. A sharp genetic discontinuity with reciprocal monophyletic lineages and limited gene flow that diverged during the middle Pleistocene was revealed between high and low latitudes. The ancestral population was small, and demographic expansions occurred in both regions during the last interglacial period. Both High- and Low-Latitude Origin scenarios were supported by approximate Bayesian computation (ABC) modeling. Ancestral area reconstruction supported the Vicariance Origin scenario. Climate analysis revealed that populations in different regions had different climatic niches and isolation by environment existed, indicating potential ecological adaptation. Ecological niche modeling indicated bidirectional expansion at present, whereas only expansion to low latitudes from high latitudes was observed in the past. Two subclusters were further revealed in both regions. Eastward and postglacial westward migrations were detected at high and low latitudes, respectively.

**Conclusions:**

The evolution of East Asian Tertiary relict species is complex, as none of the three proposed scenarios are fully supported, and the diversification within each region differs for *L. obtusiloba*. Dual refugia in high and low latitudes offer long-term survival, and populations at high latitudes may develop ecological adaptations. Genomic data with richer genetic information and adaptation loci are likely to help elucidate the origin and adaptation history of East Asian Tertiary relicts in the future.

**Supplementary Information:**

The online version contains supplementary material available at 10.1186/s12870-025-07827-6.

## Background

East Asia is characterized by high levels of biodiversity and endemism [1, 2]. Extreme physiographic and climatic heterogeneity, combined with the intensification of monsoons, provide long-term refugia for Tertiary relicts, which were once mostly widely distributed across the Northern Hemisphere [2–4]. The relict species experienced not only southward retreat during the climate cooling from the middle to late Tertiary [5, 6] but also repeated migrations northward or southward due to periodic climatic fluctuations during the Quaternary [7, 8]. Thus, their evolutionary histories are more complicated than those of species originating from low latitudes (e.g., tropical or subtropical regions).

The evolution of East Asian Tertiary relict species can be demonstrated by three distinct hypotheses. The Low-Latitude Origin (LLO) hypothesis suggests that the relict species initially diverged at low latitudes, with parts of the ancestral populations then migrating to higher latitudes [8]. A gradual decrease in genetic diversity with increasing latitude is predicted, and the genetic lineage of northern populations is nested within the southern lineage [7, 8]. Some phylogeographic case studies can be found [9, 10]. The definition and genetic predictions of the High-Latitude Origin (HLO) hypothesis are exactly the opposite of those for the LLO hypothesis. Evidence supporting the HLO scenario is primarily derived from phylogenetic studies, such as those on American oaks [11] and *Meehania* [12], while phylogeographic studies are limited, with Gugger et al. [13] being a rare example. Vicariance Origin (VCO) suggests that populations at high and low latitudes have diverged due to intermediate geographical barriers, such as the East China Sea (ECS) [8], or environmental barriers, such as the aridity belt [14]. These barriers likely result in similar levels of genetic diversity and reciprocal monophyletic lineages [8]. The dated divergence time would be older than the formation of the genetic barrier [8].

These three hypotheses can be evaluated in the context of East Asia’s two main refugial regions (NEA and SEA; see below) [14]. Understanding how Tertiary relicts are distributed across these areas allows us to test whether diversification primarily occurred in the north or south or through long-term vicariance between them. One refugia region is centered in Japan, Korea, and adjacent northeastern China (NEA), and the other is located in southern and southeastern China and extends into the Himalayas (SEA) [5]. Several Tertiary relict species (or species pairs) are distributed across both refugia regions, including *Acer pictum*, *Kalopanax septemlobus*, *Lindera obtusiloba*, *Juglans mandschrica* and *J. cathayensis*, *Euptelea pleiosperma*, and *E. polyandra*. The phylogeography of some species revealed the presence of independent clades in the NEA and SEA, with no clear pattern of “southern richness and northern poverty”. Examples include *Juglans* spp. [15], *Euptelea* [16], and *L. obtusiloba* [14]. The distinct clades can be associated with the redevelopment of the arid belt during the late Miocene to Pliocene epochs or the sea level changes of the ECS [14, 15]. Therefore, which origin scenario is the most suitable model for the evolution of the Tertiary relicts in East Asia remains unclear.

*L. obtusiloba*, a Tertiary species widely distributed in East Asia, presents a suitable case for testing the three origin hypotheses. First, our previous study revealed that the NEA and SEA *L. obtusiloba* populations were genetically isolated with limited gene flow through chloroplast DNA (cpDNA) and nuclear microsatellites (nSSRs), supporting the VCO scenario. Second, greater genetic diversity of nSSRs was detected in NEA. More abundant Miocene *L. paraobtusiloba* fossils, an analog of *L. obtusiloba* [17], have been found in NEA than in SEA, and the oldest fossil (dating back to the Paleocene) was discovered in northeastern China (approximately 50° N) [14]. Certain cold-tolerance traits, such as buds surrounded by leathery bracts [18], may have aided the species in surviving the cold climate in NEA after the Tertiary. These findings are evidence for the HLO scenario. Finally, more haplotypes and genetic diversity of cpDNA and greater morphological variation were shown in SEA [14], supporting the LLO scenario. Thus, whether the evolution of *L. obtusiloba* conforms to HLO, LLO or VCO needs further study.

In this study, we sequenced 27 nuclear low-copy genes (nLCGs) developed from transcriptome data [19]. Genetic structure and divergence time estimations were performed using Bayesian clustering and Bayesian phylogenetic inference based on the nLCGs. Ancestral area reconstruction was applied to infer possible ancestral population distributions. Different evolutionary scenarios representing HLO, LLO, and VCO with different demographic processes were designed and tested using the approximate Bayesian computation (ABC) model. Key parameters of demographic processes, such as effective population size (*N*_e_) and gene flow, were also estimated. Potential distributions and differences in niches between SEA and NEA populations were analyzed. We intend to answer the following questions: (1) Whether the extant *L. obtusiloba* populations originate from HLO, LLO, or VCO? (2) What are the detailed diversification and demographic history of *L. obtusiloba* in the two regions?

## Methods

### Sampling and DNA extraction

**Fig. 1:**
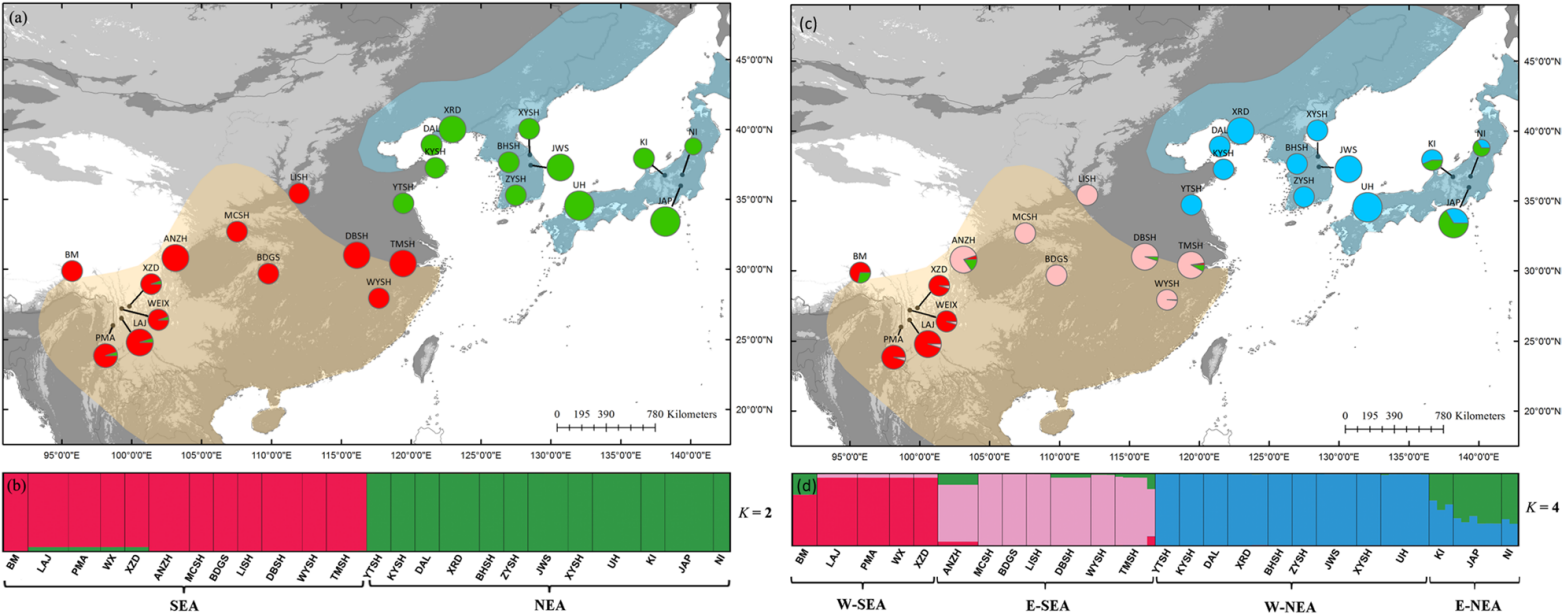
**a**, **c** Color-coded grouping of the 24 *Lindera obtusiloba* populations according to the most likely *K* = 2/4, as inferred by STRUCTURE, using 27 low-copy nuclear gene loci. **b**, **d** Histogram of the STRUCTURE assignment test for all populations at their most likely *K* = 2/4. The light orange and blue shadows represent the SEA and NEA regions of East Asian Tertiary relict flora, respectively

We sampled 90 individuals of *L. obtusiloba* from 24 populations, with 12 populations each in the NEA and SEA regions, encompassing its entire distribution range. This includes 20 populations from Ye et al.’s study [14] and four additional populations: one from Xizang (BM) in China and three from Japan (UH, KI, and NI) (Table 1; Fig. 1). To minimize the collection of closely related individuals, the sampled individuals were spaced at least 30 m apart. Samples from the related species, *L. erythrocarpa*, were collected as an outgroup. The samples were dried and stored in silica gel prior to the extraction of total genomic DNA using a plant genomic DNA extraction kit (Tiangen, Beijing). Voucher samples (BNU23236–23259), identified by Hong-Fang Wang, were deposited at the Beijing Normal University Herbarium (BNU; Table S2).

**Table 1:** Genetic diversity of 27 nuclear low-copy genes for 24 populations of *Lindera obtusiloba*

Code	Region/Population	Location	Latitude	Longitude	*n (sample size)*	Allelic richness	Nucleotide diversity (× 10^− 3^)
	SEA region				45	12.04	3.52
1	BM	Bomi, Xizang, China	29.87	95.73	3	1.64	1.00
2	PMA	Pianma, Yunnan, China	25.99	98.66	4	1.69	1.30
3	LAJ	Lajing, Yunnan, China	26.49	99.28	5	1.83	1.51
4	WEIX	Weixi, Yunnan, China	27.18	99.29	3	1.85	1.61
5	XZD	Xiaozhongdian, Yunnan, China	27.34	99.84	3	1.73	1.20
6	ANZH	Anzihe Nature Reserve, Sichuan, China	30.81	103.13	5	1.58	0.70
7	MCSH	Mt. Micang, Shannxi, China	32.69	107.53	3	1.90	1.45
8	BDGS	Mt. Badagong, Hunan, China	29.69	109.79	3	1.94	1.53
9	LISH	Mt. Li, Shanxi, China	35.43	111.98	3	1.61	0.87
10	DBSH	Mt. Daba, Anhui, China	31.01	116.11	5	1.71	1.29
11	WYSH	Mt. Wuyi, Jiangxi, China	27.93	117.69	3	1.63	1.02
12	TMSH	Mt. Tianmu, Zhejiang, China	30.42	119.41	5	1.72	1.28
	NEA region				45	6.06	1.96
13	YTSH	Mt. Yuntai, Jiangsu, China	34.72	119.44	3	1.58	1.02
14	DAL	Dalian, Liaoning, China	38.90	121.46	3	1.43	1.19
15	KYSH	Mt. Kunyu, Shandong, China	37.26	121.73	3	1.70	1.43
16	XRD	Zhuanghe, Liaoning, China	40.02	122.96	5	1.66	1.53
17	BHSH	Bukhansan National Park, Seoul City, Korea	37.65	126.99	3	1.79	1.19
18	ZYSH	Mt. Jiri, South Gyeongsang Province, Korea	35.29	127.49	3	1.71	1.54
19	XYSH	Seoraksan National Park, Gangwon Province, Korea	38.17	128.49	3	1.57	1.50
20	JWS	Gariwangsan, Gangwon Province, Korea	37.43	128.56	5	1.51	1.10
21	UH	Masuda-shi Shimane-ken, Japan	34.55	132.04	6	1.68	1.10
22	KI	Kawakami, Nagano-ken, Japan	36.73	138.15	3	1.92	1.85
23	JAP	Tokyo, Japan	35.95	139.30	6	1.89	1.81
24	NI	Nikkō-shi, Tochigi-ken, Japan	36.75	139.42	2	1.44	0.76

### Nuclear DNA

#### Sequencing and neutrality tests

Twenty-seven nLCGs were sequenced from all the sampled *L. obtusiloba* individuals (Table 1; Ye et al. [19]). In *L. erythrocarpa*, only 15 loci were successfully sequenced. CodonCode Aligner 3.6.1 (https://www.codoncode.com/aligner/) with the CLUSTAL module was used to edit and align all sequences, and the PHASE function in DnaSP 5.10.01 [20] was utilized to phase heterozygous sequences. The newly obtained nLCGs were uploaded to GenBank (MF152421–MF152590). We assessed selective neutrality using the Ewens–Watterson test, Tajima’s D, and Fu’s FS, as calculated by Arlequin 3.5 [21].

#### Genetic diversity and structure

Measures of genetic diversity, including nucleotide diversity *(π)* and haplotype diversity *(H*_d_) at the locus level, as well as genetic diversity (*π* and allelic richness, *A*_*R*_) at the population level, were calculated for the NEA and SEA regions using SPADS 1.0 [22]. The 27 loci were associated with different putative functions, as justified by the gene information of the closest gene [19]; thus, these loci were treated as independent loci in the subsequent genetic structure, phylogeny and demographic analyses. Population structure was assessed using a model-based Bayesian algorithm in STRUCTURE 2.3.4 [23] with an admixture model of population structure, and allele frequencies were assumed to be correlated among populations using a converted input file by SPADS 1.0 [22]. Ten independent runs were performed for each population (*K*) from 1 to 20 with 1 × 10^5^ Markov chain Monte Carlo (MCMC) steps of burn-in, followed by 1 × 10^6^ steps. The most likely number of clusters was determined by the change in the log-likelihood of the data, *LnP(D)*, and the second-order rate of *LnP(D)* change, Δ*K* [24]. The average membership coefficients for the 10 simulation runs of a given *K* value were generated by CLUMPP 1.1.2 [25], and a graphical representation of the average membership coefficient for each individual was generated in Distruct 1.1 [26]. The geographical distributions of different clusters were displayed in ArcMap 10.2 (ESRI, Redlands, CA, USA). Pairwise genetic differentiation (*F*_ST_) between genetic clusters was calculated using SPADS 1.0 with 1000 permutations.

#### Bayesian phylogeny and divergence time

The Bayesian phylogeny of the 24 populations and *L. erythrocarpa* was inferred using *BEAST [27] in BEAST 2.4 [28] with all partitions unlinked. The substitution models for each locus were chosen in jModelTest 2.0.1 [29]. Because no nLCG substitution rates have been published for *Lindera* or Lauraceae species, we estimated the nLCG substitution rate of *L. obtusiloba* by the suggested substitution ratio between cpDNA and nuclear DNA, which is estimated as 3:16 [30]. Based on fossil data and phylogenetic information, the cpDNA substitution rate of *L. obtusiloba* was estimated to be 8.59 × 10^− 10^, with a 95% highest probability density interval (HPD) of 3.12–23.80 × 10^− 10^ per site per year [14]. Hence, the estimated substitution rate of nLCG was 4.58 × 10^− 9^ (95% HPD: 1.66–12.69 × 10^− 9^) per site per year, which was applied to estimate coalescence time based on the Bayesian phylogeny.

The MCMC algorithm was set to run for 1 × 10^9^ steps, with sampling occurring every 1 × 10^4^ steps. The initial 20% of the samples were discarded as burn-in. The quality of the MCMC output was assessed by examining the effective sample size (ESS) value in Tracer 1.6 (http://tree.bio.ed.ac.uk/software/tracer). A maximum clade credibility tree was chosen from the sampled posterior distribution using the program TreeAnnotator 2.5.1 (part of the BEAST package). The tree topology and coalescence times were visualized in FIGTREE 1.4.2 (http://tree.bio.ed.ac.uk/software/figtree). Multiple runs of combinations of different clock models (strict clock or lognormal clock model) and different coalescent priors (Yule prior or coalescent prior) resulted in similar divergence times for all populations, the SEA or NEA populations, and the results with strict clock models and the Yule process were reported.

#### IMa analysis

We utilized the ‘isolation with migration’ (IM) coalescent model, as implemented in IMa2 [31], to estimate the *N*_e_ of NEA, SEA, and their common ancestral populations, the bidirectional migration rates (2*Nm*) and the divergence time (*t*) between the two regions. All the loci were analyzed using the HKY model, and the inheritance scalars were set to 1. The corresponding values of u were calculated using the formula u = *µ*kg, where *µ* represents the substitutions per site per year (4.58 × 10^− 9^, 95% HPD: 1.66–12.69 × 10^− 9^), *k* is the sequence length of each locus, and g is the generation time, which is approximately 15 years in *L. obtusiloba* [14].

MCMC simulations were conducted using a geometric heating scheme (with the required two terms set to 0.99 and 0.75) across 60 chains. The burn-in phase was run for a sufficient duration to ensure that no perceivable trends were present in the trend plots. Subsequently, 2 × 10^6^ steps were executed to save 2 × 10^4^ genealogies. The posterior Hipt (highest bin value) and 95% HPD for the demographic parameters are reported. Only estimates whose posterior distribution fell to zero within the prior intervals were considered reliable. All individuals were included, and two independent runs were performed. The results of the second run are reported.

#### MsABC modeling

To determine whether *L. obtusiloba* originated through HLO, LLO, or VCO, the approximate Bayesian computation (ABC) algorithm was employed using msABC [32]. A split model was defined as the derived population formed from an expansion of a fraction of the ancestral population with minimal or no change in population size. In contrast, the vicariance model assumes that the population size of the ancestral populations is the sum of all derived populations. Six split and nine vicariance scenarios with different demographic parameters were constructed (see supporting file 1).

After simulation of 2 × 10^6^ steps in each model, model comparison and parameter estimation were conducted in the statistics package ‘abc’ in R 3.5.0 (https://www.r-project.org/). The multinomial logistic regression method (“mnlogistic” function) was used to infer the posterior probability of each demographic model with a tolerance rate of 0.01. For the best-supported model, the divergence time, *N*_e_ and gene flow were estimated using Neuronet’s method with nnet = 50. The median value and 95% HPD of all the estimations are reported. Two independent runs were conducted for msABC simulations, model selection and parameter estimation to ensure congruence, and the results of the second run are reported.

#### Ecological niche modeling and climatic differences

Ten uncorrelated climatic variables (*r* < 0.9 [14]), were used to model the current niches of the NEA (35 occurrences) and SEA (65 occurrences) populations through the maximum-entropy modeling technique (MaxEnt [33]). Potential distributions at the Last Glacial Maximum (LGM) were predicted with MIROC-ESM, an Earth System Model based on the Model for Interdisciplinary Research on Climate [34] and CCSM4, a Community Climate System Model, version 4 [35]. In ENMTools, a background test with 1000 pseudoreplicates based on the 10 climatic variables was used to test whether the ecological niche models (ENMs) generated from the two regions were identical [36]. Tests were performed in both directions (NEA *vs*. SEA and vice versa) by taking background samples from buffer zones (0.5°), defined based on their dispersal ability [37]. Niche similarity was quantified using both Schoener’s *D* and the standardized Hellinger distance (*I*) [36].

Nineteen climate variables, downloaded from WorldClim [38] at a 2.5-arcminute resolution, were extracted using Spatial Analyst Tools in ArcGIS 10.2 (ESRI, Redlands, CA, USA). A principal component analysis (PCA, using the ‘princomp’ function) was conducted in R. Isolation by environment (IBE) was assessed through partial Mantel tests (‘mantel.partial’ function) between the *F*_ST_ and climatic (Euclidean) distance, accounting for geographic distance in the form of a natural logarithm, which was based on 10,000 permutations. These analyses were conducted across all the populations, as well as in the SEA and NEA populations.

#### Ancestral area reconstructions

To reconstruct the geographical diversification, five geographic regions, Eastern Himalayan Province (A), Sikang–Yunnan Province (B), Central Chinese Province (C), North Chinese Province (D) and Japanese–Korean Province (E), were defined according to the floristic divisions [1]. BioGeoBEARS 1.1.1 [39] in R was applied based on the dated Bayesian phylogeny. An equal probability for exchange between different areas was applied, and the maximum number of areas was assigned to five. Six models (DEC, DEC + j, DIVALIKE, DIVALIKE + j, BAYAREALIKE, and BAYAREALIKE + j) were generated and compared based on the AICc weights.

## Results

### Genetic diversity and genetic structure

No loci significantly violated the neutrality assumption (Table S1). The sequence length ranged from 154 to 944 base pairs (bp), totaling 13,285 bp; the number of variable sites ranged from three to 71; *H*_d_ ranged from 0.56 to 0.97; and *π* ranged from 1.6 × 10^− 3^–8.56 × 10^− 3^ (Table S1). Among the 24 populations, the *A*_R_ ranged from 1.44 to 1.90, and the *π* ranged from 0.70 × 10^− 3^–1.85 × 10^− 3^ (Table 1). Genetic diversity in the SEA region (*A*_R_ = 12.04, *π* = 3.52 × 10^− 3^) was almost twice that in the NEA region (*A*_R_ = 6.06, *π* = 1.96 × 10^− 3^), with an equal sample size (*n* = 45).

**Fig. 2:**
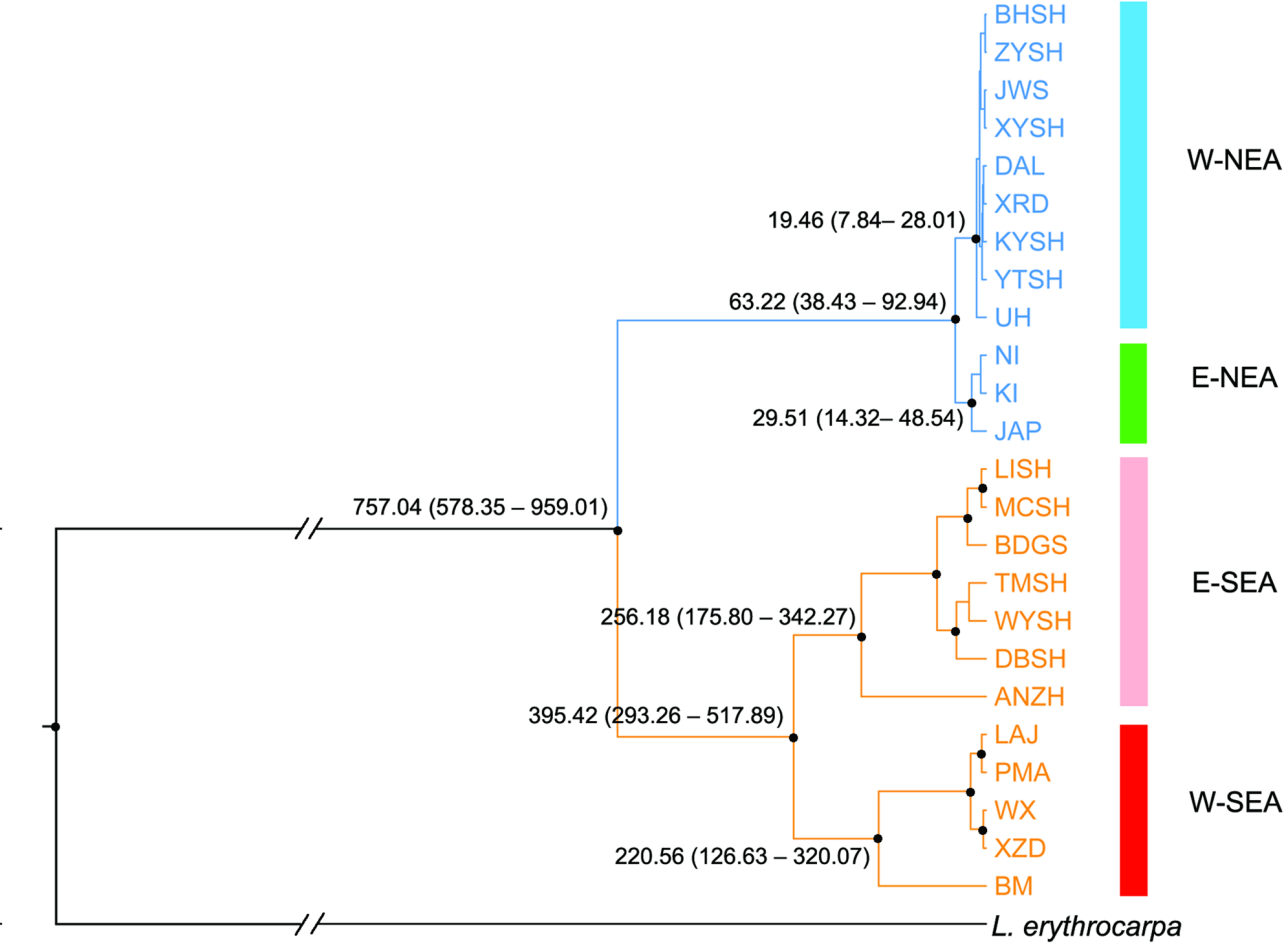
BEAST-derived chronograms of 24 populations of *Lindera obtusiloba* with outgroup *L. erythrocarpa* using 15 low-copy nuclear gene loci (the remaining 12 loci were not successfully sequenced in *L. erythrocarpa*) based on the estimated substitution rate, mean: 4.58 × 10^− 9^ (95% highest-probability-density interval, HPD, 1.66–12.69 × 10^− 9^). Black dots in nodes indicate high posterior probabilities (PP > 0.95). Median divergence time and 95% HPD in key nodes are labeled; all estimated divergence times were scaled in ka, a thousand years ago. Clades and populations in the NEA and SEA regions are colored in blue and orange, respectively. Four clusters in accord with STRUCTURE results (see Fig. 1) are shown in the same color

In the STRUCTURE analysis, Δ*K* had the highest value when *K* = 2 (Fig. S2), with the two clusters corresponding to the NEA and SEA division (Fig. 1a). The second highest Δ*K* occurred when *K* = 4, and *lnP*(D) also almost reached the highest value (Fig. 1b and S1). The four clusters formed four reciprocally monophyletic lineages (PP > 0.95) in the BEAST-derived phylogeny (Fig. 2). Greater genetic differentiation was detected between the two clusters in SEA (*F*_ST_ = 0.54) than between the two in NEA (*F*_ST_ = 0.39), and the greatest genetic differentiation was detected between clusters from NEA and SEA (*F*_ST_ = 0.75–0.79) (Table 2).

**Table 2:** Pairwise *F*_ST_ values between all four clusters of* Lindera obtusiloba* derived from Bayesian clustering and phylogeny using 27 nuclear low-copy genes

Clusters	W-SEA	E-SEA	W-NEA	E-NEA
W-SEA	0.00			
E-SEA	0.54	0.00		
W-NEA	0.78	0.79	0.00	
E-NEA	0.75	0.77	0.39	0.00

### Population demographic history

BEAST analysis revealed that the coalescence time for all the populations was 757.04 ka (95% HPD: 578.35–959.01 ka), with the populations in NEA and SEA coalescing at 63.22 ka (95% HPD: 38.43–92.94 ka) and 395.42 ka (95% HPD: 293.26–517.89 ka), respectively (Fig. 2). The IMa2 estimated a coalescence time at 798.61 ka (95% HPD: 577.16–96.49 ka). The estimated gene flow was low (*Nm* < 0.01), and the effective population size of SEA populations (*N*_SEA_ = 2.4 × 10^4^) was approximately three times that of NEA populations (*N*_NEA_ = 0.9 × 10^4^) and ten times that of ancestral populations (Table 3 and Fig. S3).

**Table 3:** Posterior Hipt (the Bin with the highest value) and 95% highest posterior density interval (HPD) of divergence time (t), effective population size and population migration rates (2Nm) estimations simulated by isolation by migration (IM) model using 27 nuclear low-copy genes of Lindera obtusiloba. ka, a thousand years ago

Parameter	t (ka)	Effective population size				Population migration rates	
		SEA	NEA	Ancestral		SEA→NEA	NEA→SEA
HiPt	798.61	23,636	8,873	3,377		0.001	0.017
95% HPD low	577.16	20,024	6,518	236		0	0.002
95% HPD high	996.49	27,720	11,386	9,659		0.029	0.052

**Fig. 3:**
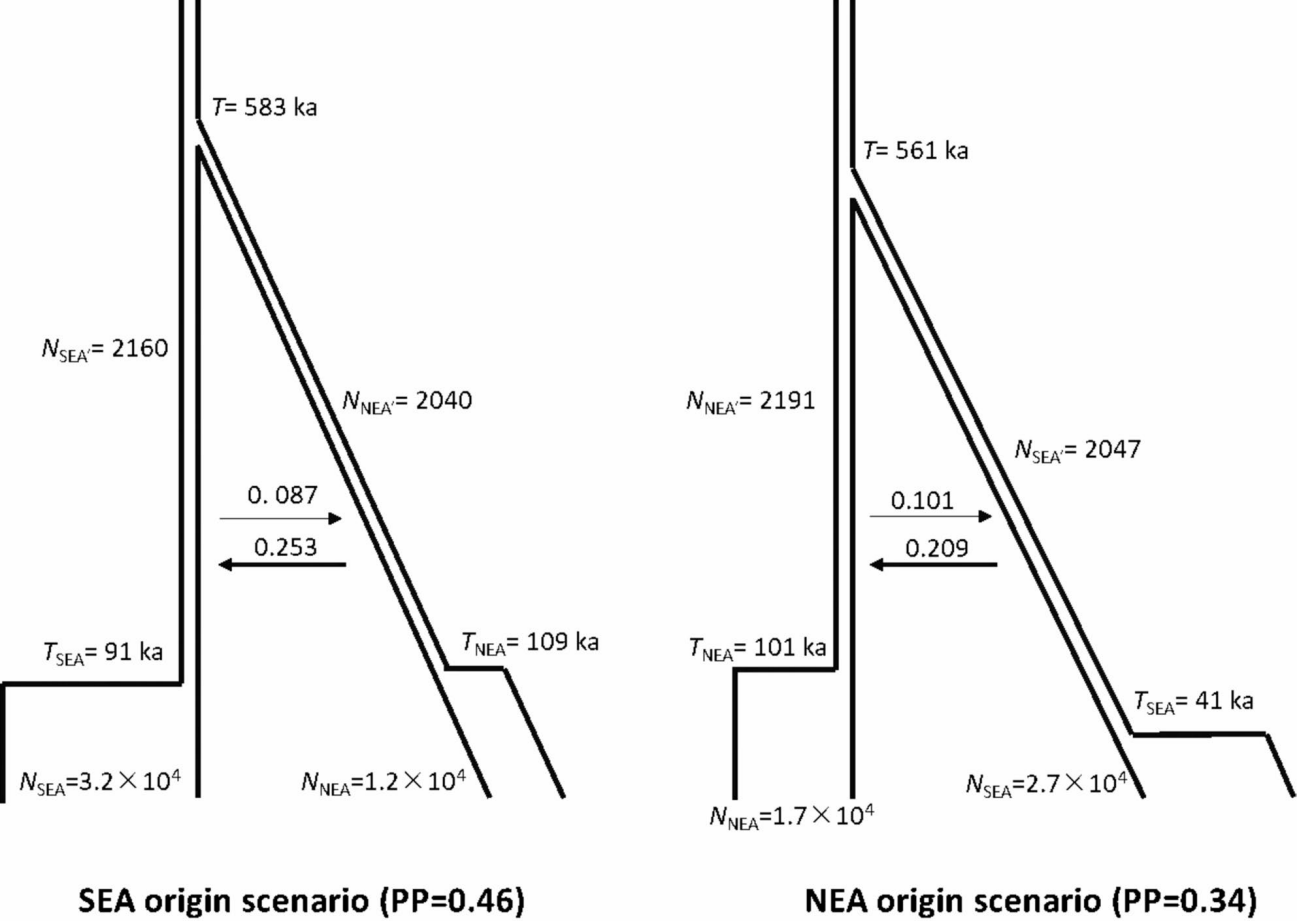
The two most possible scenarios, SEA origin (**a**) and NEA origin (**b**) in msABC. Posterior probabilities (PP) in different scenarios are shown. Divergence times (*T*, *T*_SEA_ and *T*_NEA_) are scaled in ka, a thousand years ago. Gene flow (*Nm*) across the divergence history is labeled. Effective population sizes of ancestral population (*N*_SEA’_ and *N*_SEA’_) and extant populations (*N*_SEA_ and *N*_SEA_) are shown

MsABC exhibited a high posterior probability of originating from SEA (PP = 0.46) or NEA (PP = 0.34), whereas the other 13 models had probabilities below 0.04. Similar demographic parameters were estimated in the two scenarios. The effective population size of SEA (3.2 × 10^4^) was greater than that of NEA (1.2 × 10^4^), and both populations experienced severe shrinkage to similar sizes (approximately 2000) at similar ages (109 ka in NEA and 91 ka in SEA) after coalescence at 583 ka. Greater migration (*Nm*) from NEA to SEA (0.25 *vs*. 0.09) was estimated in the SEA origin scenario, whereas the opposite migration (0.21 *vs*. 0.10) was greater in the NEA origin scenario (Fig. 3 and Figs. S4-5).

### Climatic-induced differences

In the PCA, the first two axes accounted for 67.7% of the total variance, with Axis 1 explaining 41.8% and Axis 2 explaining 25.9% (see Table S2). PC1 and PC2 are primarily associated with precipitation and temperature, respectively (Table S2). The data in Fig. 5a suggest that populations from SEA and NEA inhabit distinct environmental and climatic niches, with the niches of the former being significantly larger. The IBE indicates potential ecological adaptation in PC2 (*r* = 0.22, *P* = 0.01), whereas there is no such indication in PC1 (*r* = 0.02, *P* = 0.22). Analyses using the 19 variables suggest potential adaptations to both precipitation and temperature (Table S3).

**Fig. 4:**
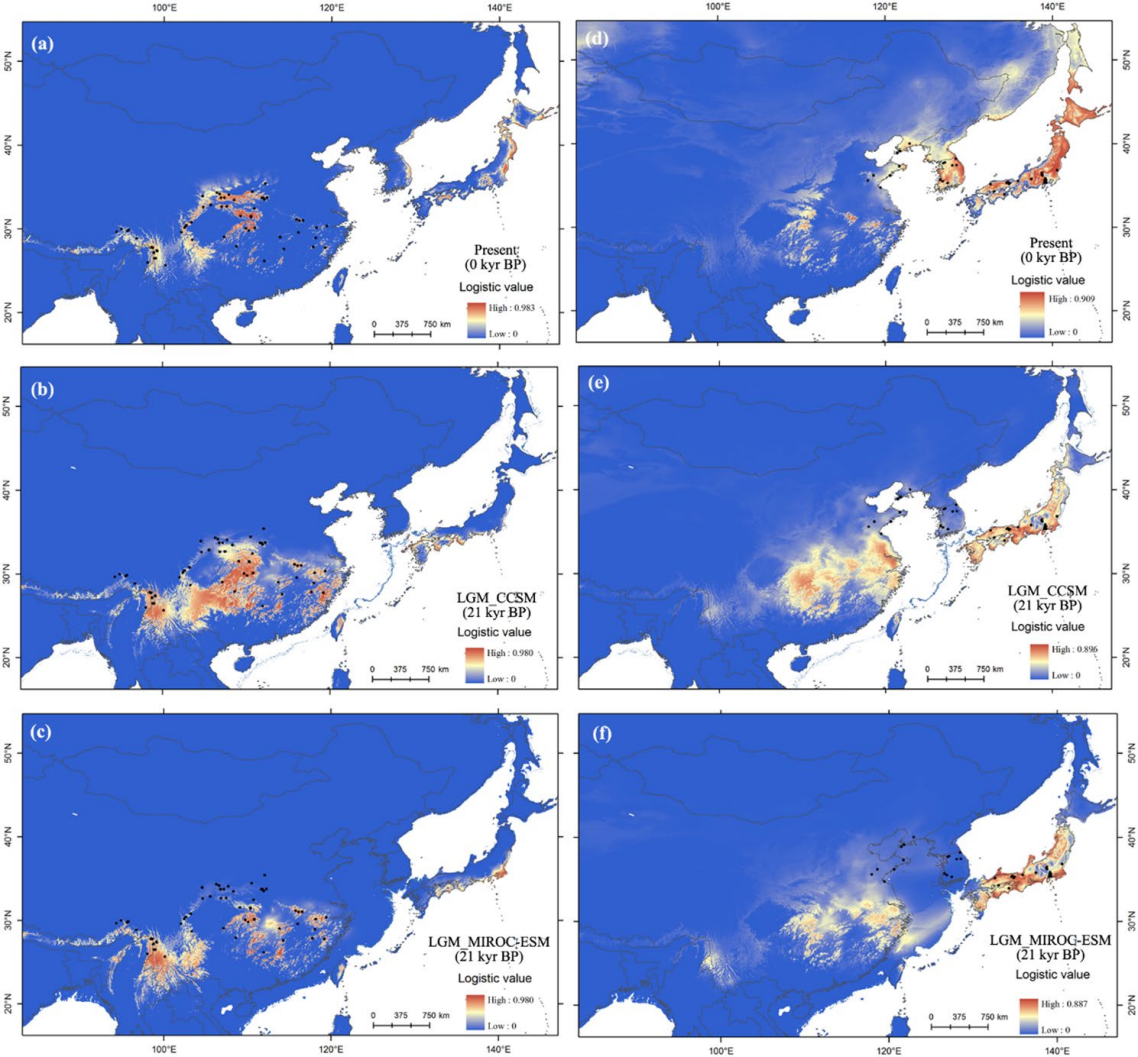
Potential species distributions modelled through ten uncorrelated climatic variables (*r* < 0.9 [14]), of SEA (**a**-**c**) and NEA (**d**-**f**) populations of *Lindera obtusiloba* based on ecological niche modelling at present (**a**, **d**), during the Last Glacial Maximum using the CCSM4 model (**b**, **e**), and the MIROC-ESM model (**c**, **f**)

**Fig. 5:**
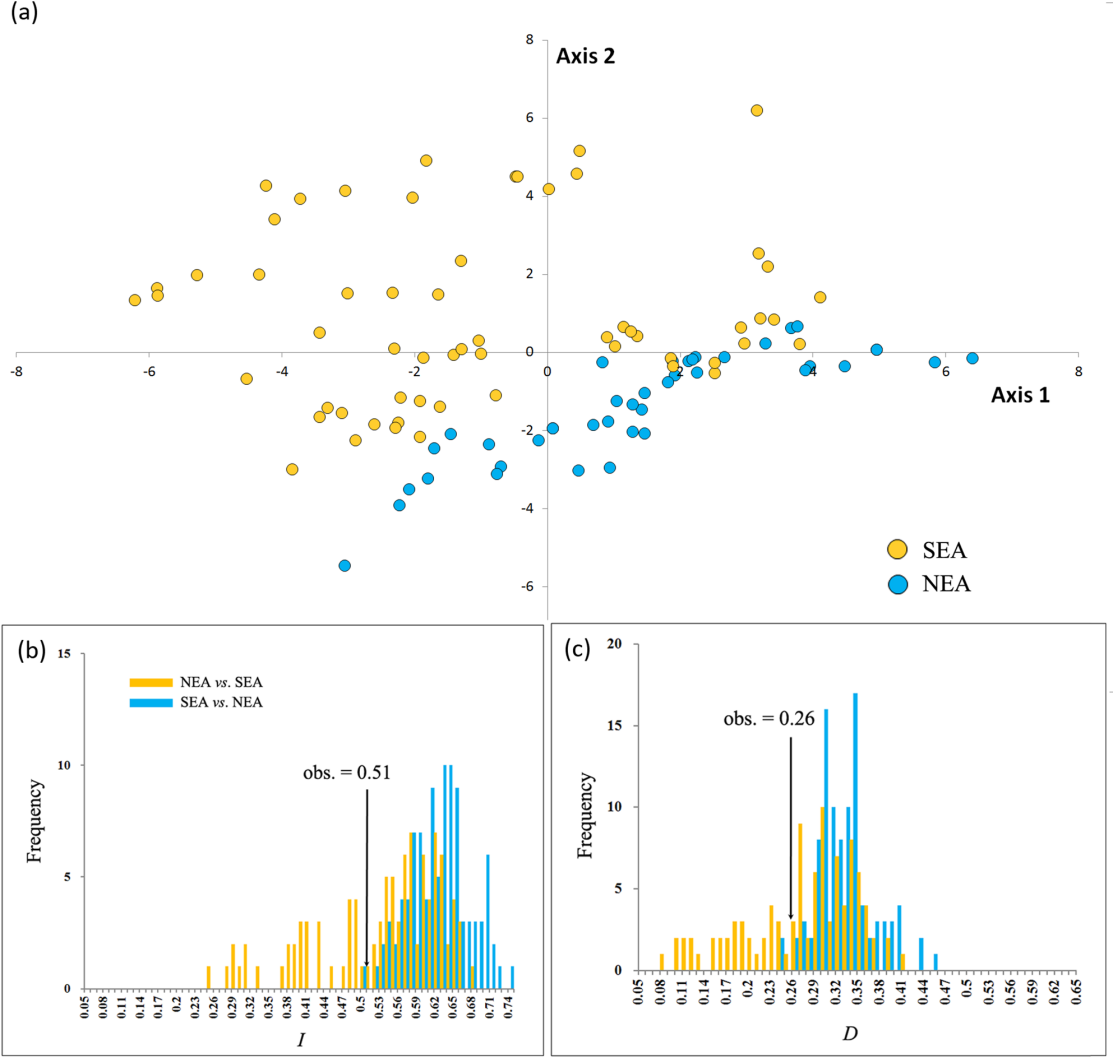
**a** Principal component analysis (PCA) plots with 19 climatic variables (Table S2) of 100 *Lindera obtusiloba* occurrence points. Different colors correspond to the SEA and NEA region. **b**, **c** Niche background test plots for 10 non-correlated climatic variable between populations from NEA and SEA regions quantified by the standardized Hellinger distance (*I*) and Schoener’s *D* [36]. The vertical line in each plot represents the observed values (obs.) while the histograms represent those of null distributions

At present, the potential distribution in both regions suggests possible extensions into the other region. In NEA, extensive suitable habitat is predicted in the northern Japanese archipelago. During the LGM, the potential distribution of the NEA populations extended into SEA regions, whereas the SEA population retracted to lower latitudes (Fig. 4). Background tests indicated that the environmental conditions in the two regions were more divergent than anticipated (*P* < 0.05) when the habitat available to NEA populations is considered. In contrast, this divergence was not observed based on the availability of SEA populations (Figs. 5b&c).

### Ancestral area reconstructions

BioGeoBEARS revealed that the DEC + j model best explained the divergence of *L. obtusiloba* populations based on AICc (DEC = 53.4, DEC + j = 43.6, DIVALIKE = 49.9, DIVALIKE + j = 45.3, BAYAREALIKE = 84.7, and BAYAREALIKE + j = 50.1). The model suggested that the ancient (mid–Pleistocene) distribution of *L. obtusiloba* was probable in the BE (Sikang–Yunnan Province and the Korean peninsula and Japanese archipelago). A vicariance event between NEA (E) and SEA (B) occurred. Within SEA, the ancestral area was likely in southwest China (B), and colonization from southwest China (B) to the Eastern Himalayan Province (A) and Central Chinese Province (C) occurred. In NEA, the ancestral area was likely in the Korean peninsula and Japanese archipelago (E), and ancestral populations experienced westward migrations to the North Chinese Province (D) (Fig. S6).

## Discussion

### The complex origin history of *L. obtusiloba* in East Asia

To test which of the three hypotheses—High-Latitude Origin (HLO), Low-Latitude Origin (LLO), or Vicariance (VCO)—best explains the evolutionary history of Tertiary relict species in East Asia, we reconstructed the phylogeography of *L. obtusiloba* through ample nuclear gene- and climate-induced difference analyses. Overall, our results are most consistent with dual refugia and asymmetric persistence, with SEA acting as a long-term demographic reservoir and NEA persisting in smaller, environmentally constrained pockets rather than a single, unambiguous origin.

In SEA, a much greater current effective population size and genetic diversity were revealed than in NEA, consistent with the higher cpDNA diversity [14], supporting the LLO pattern [7, 8]. The SEA origin scenario in the MsABC approach also received the highest support. The environments in the two regions were more divergent than expected based on the habitats available to the NEA populations, indicating that *L. obtusiloba* preferred a humid and warm climate in subtropical regions [40]. HLO is supported mainly by fossil evidence. *L. paraobtusiloba*, a fossil species that closely resembles *L. obtusiloba*, had its oldest occurrence in NEA (dated to the Paleocene, approximately 50° N) and its youngest one in SEA (Yunnan Province, approximately 25° N) [14]. In the Miocene, more fossils were found in NEA than in SEA [14]. The potential distribution modelling also show the present NEA populations has possibilities to colonize northern Japanese archipelago with severer environment. The NEA origin scenario in msABC (PP = 0.34) provides additional modest support. The higher genetic diversity of nSSRs in the NEA region may have been caused by a limited number of loci (*n* = 6) [14]. However, the Bayesian phylogeny revealed two reciprocal monophyletic lineages, with no nested relationships, favoring the VCO scenario [8]. Ancestral area reconstruction further supported the VCO pattern, indicating long-term persistence in dual refugia (SEA and NEA regions). The best fit model (DEC + j) indicates that the founder-event process is also included, which are likely occurred in demographic history within the two regions [39]. The ABC model did not find evidence of population shrinkage during the LGM, which further supports that the NEA and SEA regions acted as dual refugia for *L. obtusiloba*. In NEA, cold tolerance characteristics, such as heavier seeds and later-maturing fruits [14], provide the possibility of long-term northern persistence [18] or may imply adaptations to the cooler climate (IBE analysis) of *L. obtusiloba* to the limited niche.

Although the three origin hypotheses are conceptually mutually exclusive, ancient divergence and subsequent asymmetric evolution with small effective population sizes (approximately 2000) may obscure clear genetic signals for dispersal direction (HLO, LLO, or VCO). Migrations between the NEA and SEA regions are likely facilitated by the exposure of the ECS seafloor since the late Miocene: 7.0–5.0 Ma, 2.0–1.3 Ma [16, 41], as the arid belt likely acted as a climatic barrier for genetic exchanges [14, 15]. Although a final ECS exposure occurred from 0.2 to 0.015 Ma [41], intensified aridity after the middle Pleistocene and possible adaptation to the environment of NEA populations may have prevented migration through the ECS [14], leading to long-term independent evolution of NEA and SEA populations.

Compared with our previous study, in which cpDNA (approximately 3 kb) and nSSRs (*n* = 6) were used [14], the 27 unlinked loci [19] provide much more information. The nLCG data reveal not only a sharp NEA–SEA genetic discontinuity but also a clear substructure and more detailed population demographic histories within each region (Figs. 1 and 2). However, the total length of nLCGs (approximately 13 kb) represents limited coverage of the total nuclear genome [42], limiting the ability to distinguish different evolution models (HLO, LLO and VCO).

### Detailed diversification history of *L. obtusiloba*

The nuclear genes traced the divergence of NEA and SEA populations to the mid–Pleistocene Transition period (MPT, 0.7–1.25 Ma) [43], whereas Ye et al. [14] reported that divergence occurred during the late Pliocene using cpDNA and nSSRs. The limited migration of cpDNA (via seeds) compared with that of nuclear genes (via seeds and pollen) should result in a larger effective population size and thus a longer coalescence time [44]. Incomplete lineage sorting and randomness in the coalescence process will result in serious overestimation of the divergence time in a gene tree (cpDNA) compared with that of a species tree (multiple nLCGs) [44]. Uncertainty in mutation models of nSSRs [44] and estimations without considering gene flow may cause an earlier estimate of the divergence time in nSSRs [14]. The MPT was characterized by alternation from mostly symmetric cycles with periods of approximately 41 ka to strongly asymmetric 100-ka cycles [43], and changes in climate may have isolated populations in various refugia, leading to divergence between NEA and SEA populations [45].

The SEA mountainous regions provided long-term refugia for Tertiary relict species [46]. Greater physiographic and climatic heterogeneity in SEA offered wider environmental niches (Fig. 5), combined with the intensification of monsoons in SEA, resulting in greater genetic diversity and a longer evolutionary history in SEA than in NEA [2–4]. Isolation in different mountains also caused greater genetic differentiation and more detailed genetic structures [14]. The detailed west–east substructure corresponds well to the Tanaka–Kaiyong Line, an important phytogeographic boundary in Southwest China [47]. Ancestral populations have survived in the Hengduan Mountains, and populations in the westernmost and central–east regions have been formed through migrations of these ancestral populations, which is consistent with the eastward migration revealed by the cpDNA and nSSR data [14].

In NEA, *L. obtusiloba* survived the unsuitable climate during the LGM in the Korea Peninsula and Japanese Archipelago. Long-term survival in the NEA region has also been reported for Asian butternuts [15], *Cercidiphyllum* [48], and *Euptelea* [16], indicating that northern migrorefugia are shared among different Tertiary relict species [8]. Ancestral area reconstruction revealed that populations in North China are formed through eastward postglacial migrations, and the gradually decreased cpDNA haplotype diversity with decreasing longitude provide further evidence [14]. However, compared with the SEA region, a more severe climate and a narrower niche would cause a faster loss of alleles, resulting in a shorter evolutionary history and lower genetic diversity [7, 8]. Lower genetic differentiation with ambiguous genetic substructure (Figs. 1 and 2) may be caused by lower physiographical and climatic heterogeneity and higher connectivity [8].

In the NEA and SEA regions, demographic expansions were both found after the last interglacial, which is in line with the prediction of glacial contraction and interglacial expansions during the Quaternary [7, 8]. Most relict species experienced multiple cycles of demographic expansion and contraction during the late Tertiary/Quaternary periods, e.g., *Tetracentron sinense* [49], *Davidia involucrata* [50], and *Ginkgo biloba* [51], whereas some species continued to decline after the LGM [50, 51]. *L. obtusiloba* populations in both SEA and NEA remained stable during and after the LGM, suggesting that Quaternary climate fluctuations, particularly the LGM, had a minor effect on their demographic histories.

## Conclusions

The evolution of East Asian Tertiary relict species is complex, as none of the three proposed origin scenarios are fully supported, and the diversification patterns vary between regions in *L. obtusiloba*. Analyses based on a limited number of neutral nuclear loci (27 loci, approximately 13 kb) may limit the ability to distinguish complex demographic scenarios with low levels of migration. Genomic data with richer genetic information and adaptation loci are likely to help elucidate the origin and adaptation history of *L. obtusiloba*. Some other Tertiary relict species also exhibit independent long-term persistence in northern populations, e.g., Asian butternuts [15], *Cercidiphyllum* [48], and *Euptelea* [16]. An integrated study of these species will lead to a deeper understanding of the complex evolutionary history of East Asian Tertiary relict species.

## Supplementary Information

Supplementary Material 1. Supporting file S1 Different origin scenarios simulated in msABC and according codes.

Supplementary Material 2. Table S1 Genetic diversity and neutrality test in 27 nuclear low-copy genes of *Lindera obtusiloba*. Figure S1 The five major floristic divisions, Eastern Himalayan Province (A), Sikang–Yunnan Province (B), Central Chinese Province (C), North Chinese Province (D) and Japanese–Korean Province (E), in East Asia according to Wu and Wu [1]. Figure S2 Delta-K and LnP(D) values from the STRUCTURE analysis on 24 *Lindera obtusiloba* populations using 27 nuclear low-copy genes with predefined group number *K* = 1–20. Standard deviations of LnP(D) obtained from 10 independent runs for each group number are also shown. Figure S3 IMa2 analysis results of *Lindera obtusiloba* populations using 27 low-copy nuclear genes. Posterior probability distributions of divergence time (t) between NEA and SEA populations (a) and effective population size of NEA, SEA and Ancestral populations (b) are illustrated. Figure S4 Posterior probability distributions of estimated demographic parameters in SEA origin (expansion) scenario. 1 and 2 represent populations in SEA and NEA, respectively. Figure S5 Posterior probability distributions of estimated demographic parameters in NEA origin (expansion) scenario. 1 and 2 represent populations in SEA and NEA, respectively. Table S2 Voucher numbers for 24 sampled populations of *Lindera obtusiloba*. Table S3 Bio-climatic variables, standardized loading for the two first axes of the principal component analysis (PCA) (present climate) and result of isolation by environment (IBE) analysis. Figure S6 Ancestral area reconstructions derived by DEC+j model usging BioGeoBEARS base on the BEAST-derived chronograms of 24 populations of *Lindera obtusiloba* with outgroup *L. erythrocarpa* (ZHGS) using 15 low-copy nuclear gene loci (the remaining 12 loci were not successfully sequenced in *L. erythrocarpa*) based on the estimated substitution rate, mean: 4.58 × 10-9 (95% highest-probability-density interval, HPD, : 1.66–12.69 × 10-9). Five geographic regions, Eastern Himalayan Province (A), Sikang–Yunnan Province (B), Central Chinese Province (C), North Chinese Province (D) and Japanese–Korean Province (E), were defined according to the floristic divisions [1].

## Data Availability

The newly obtained nLCGs were uploaded to GenBank (MF152421-MF152590).
