# Supplementary material for: Complex evolution of East Asian Tertiary relict species revealed by the phylogeography of Lindera obtusiloba
Source: BMC Plant Biol. 2025 Dec 9;26:74. doi: 10.1186/s12870-025-07827-6 (PMC12801852; doi:10.1186/s12870-025-07827-6)
Supplement: Supplementary file 1 — Supplementary Material 1. Supporting file S1 Different origin scenarios simulated in msABC and according codes. [file 12870_2025_7827_MOESM1_ESM.pptx]

## Slide 1
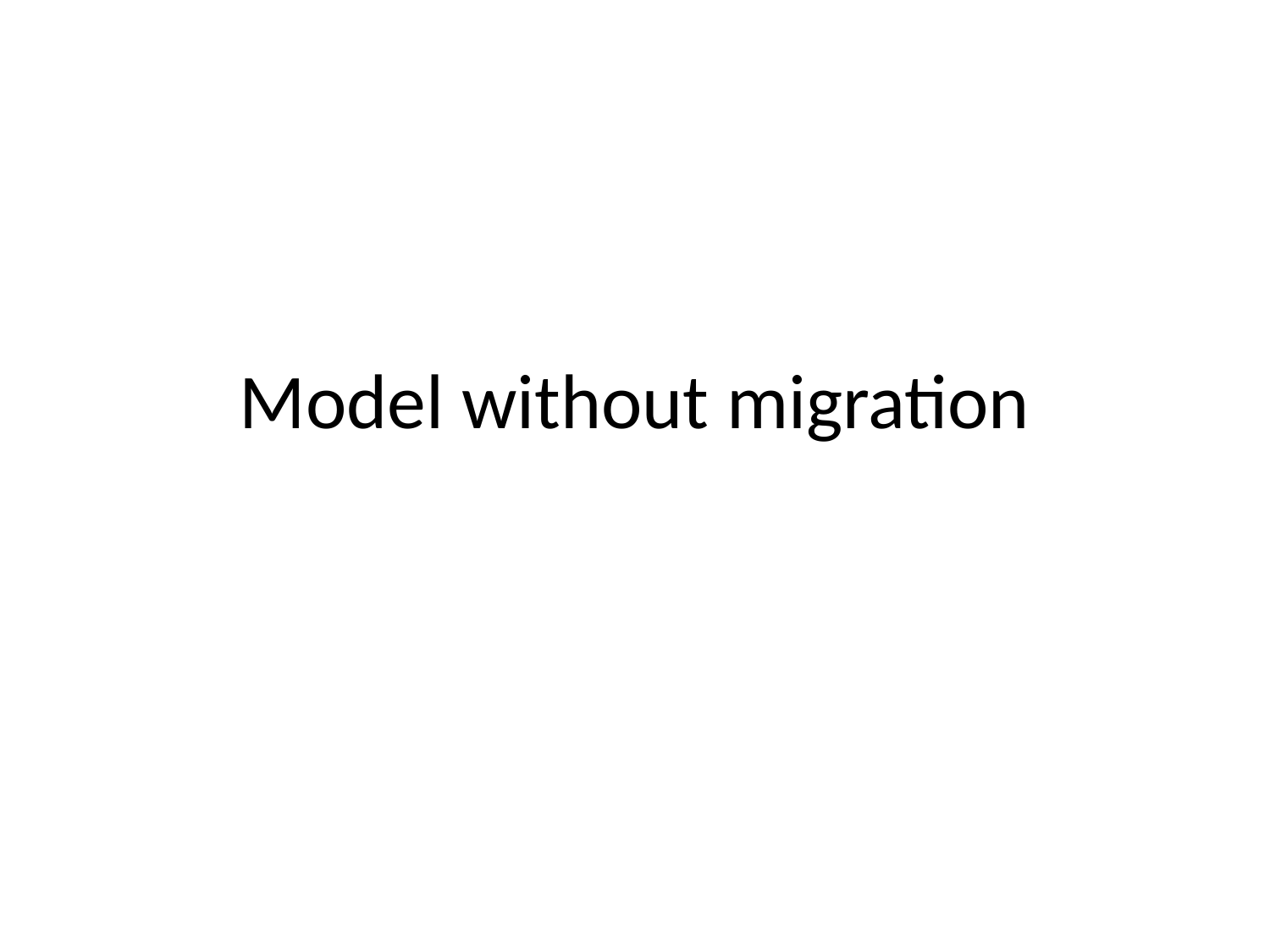

# Model without migration

## Slide 2
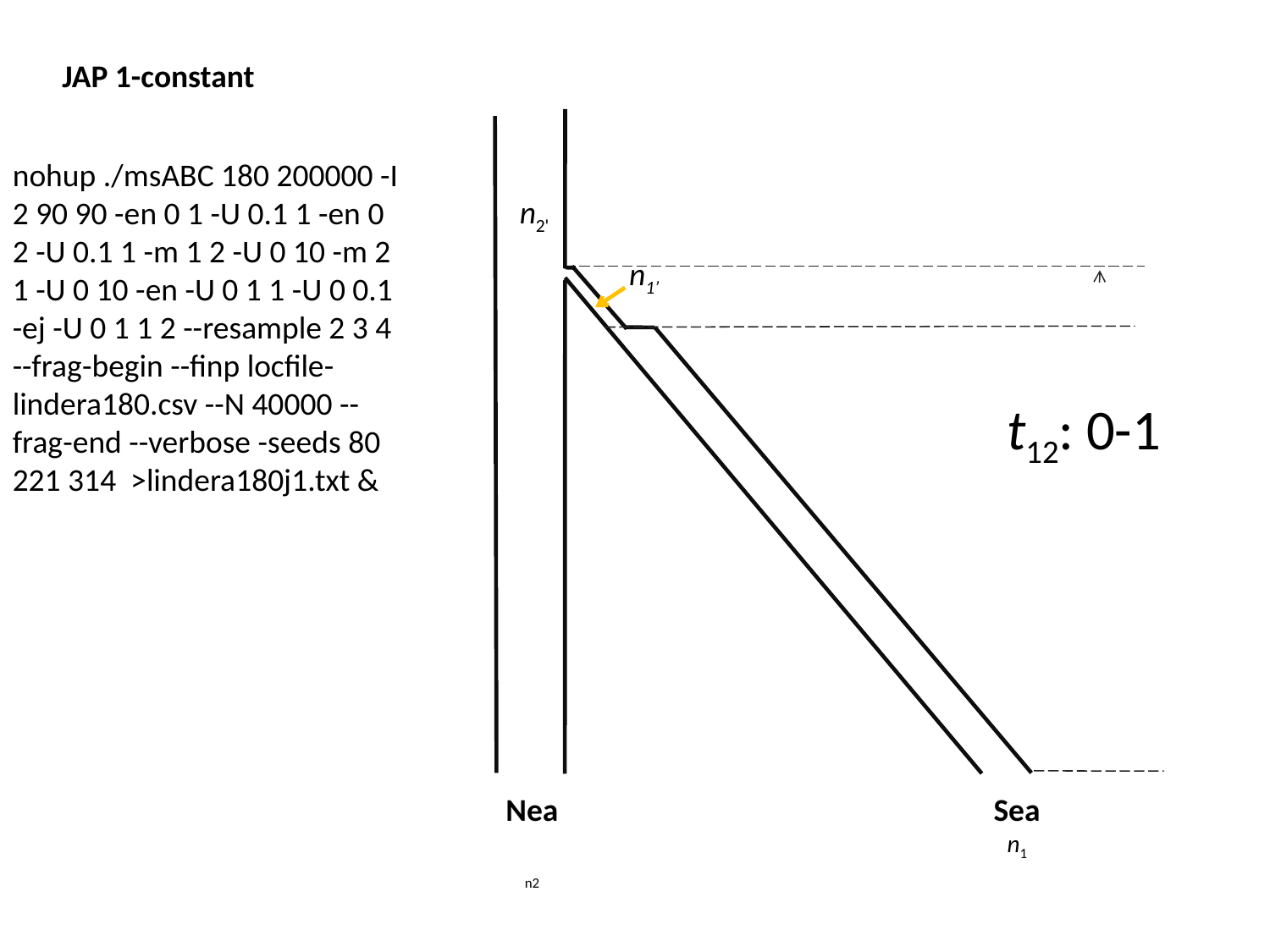

JAP 1-constant
nohup ./msABC 180 200000 -I 2 90 90 -en 0 1 -U 0.1 1 -en 0 2 -U 0.1 1 -m 1 2 -U 0 10 -m 2 1 -U 0 10 -en -U 0 1 1 -U 0 0.1 -ej -U 0 1 1 2 --resample 2 3 4 --frag-begin --finp locfile-lindera180.csv --N 40000 --frag-end --verbose -seeds 80 221 314 >lindera180j1.txt &
n2'
n1’
t12: 0-1
Nea
n2
Sea
n1

## Slide 3
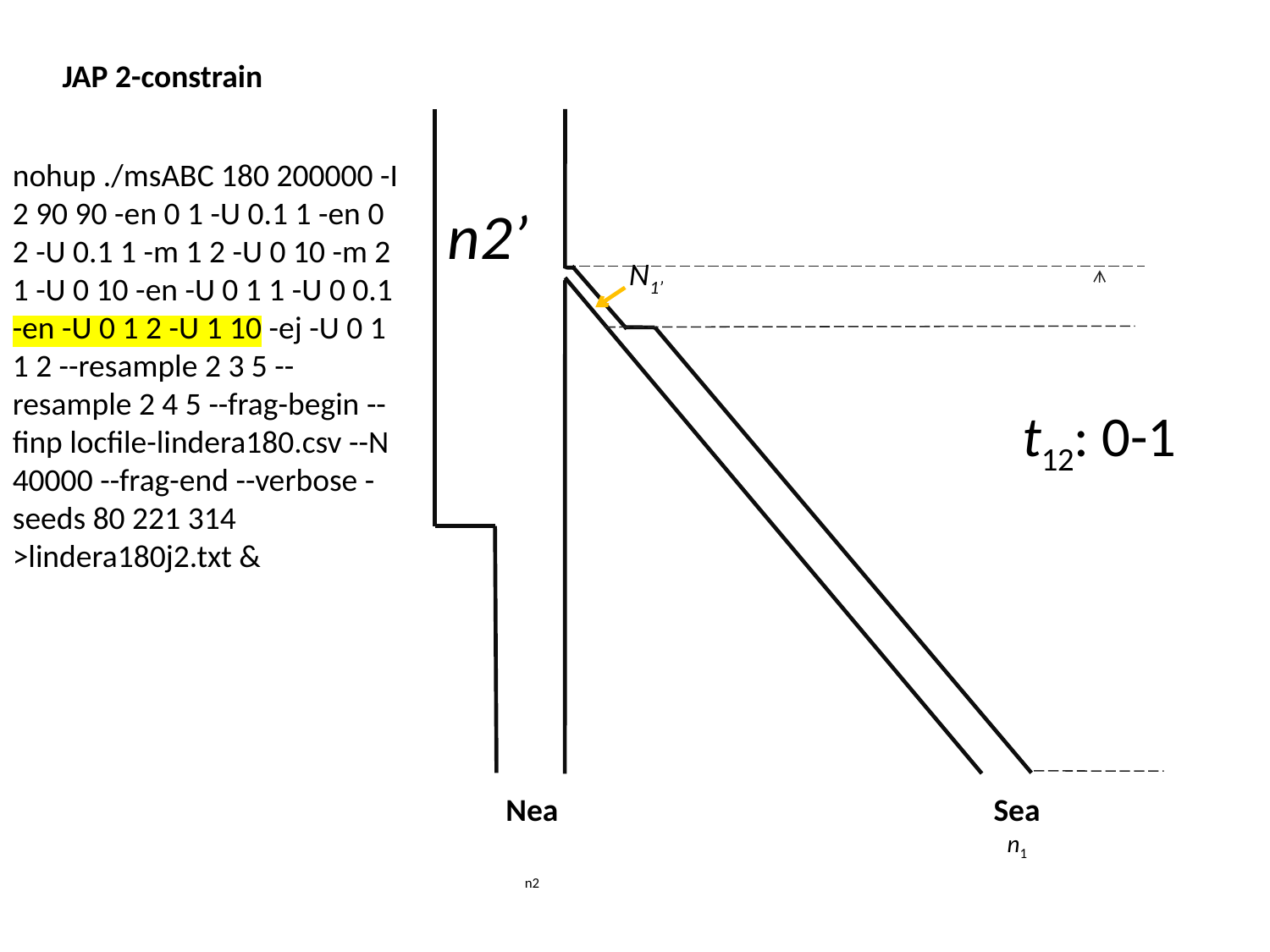

JAP 2-constrain
nohup ./msABC 180 200000 -I 2 90 90 -en 0 1 -U 0.1 1 -en 0 2 -U 0.1 1 -m 1 2 -U 0 10 -m 2 1 -U 0 10 -en -U 0 1 1 -U 0 0.1
-en -U 0 1 2 -U 1 10 -ej -U 0 1 1 2 --resample 2 3 5 --resample 2 4 5 --frag-begin --finp locfile-lindera180.csv --N 40000 --frag-end --verbose -seeds 80 221 314 >lindera180j2.txt &
n2’
N1’
t12: 0-1
Nea
n2
Sea
n1

## Slide 4
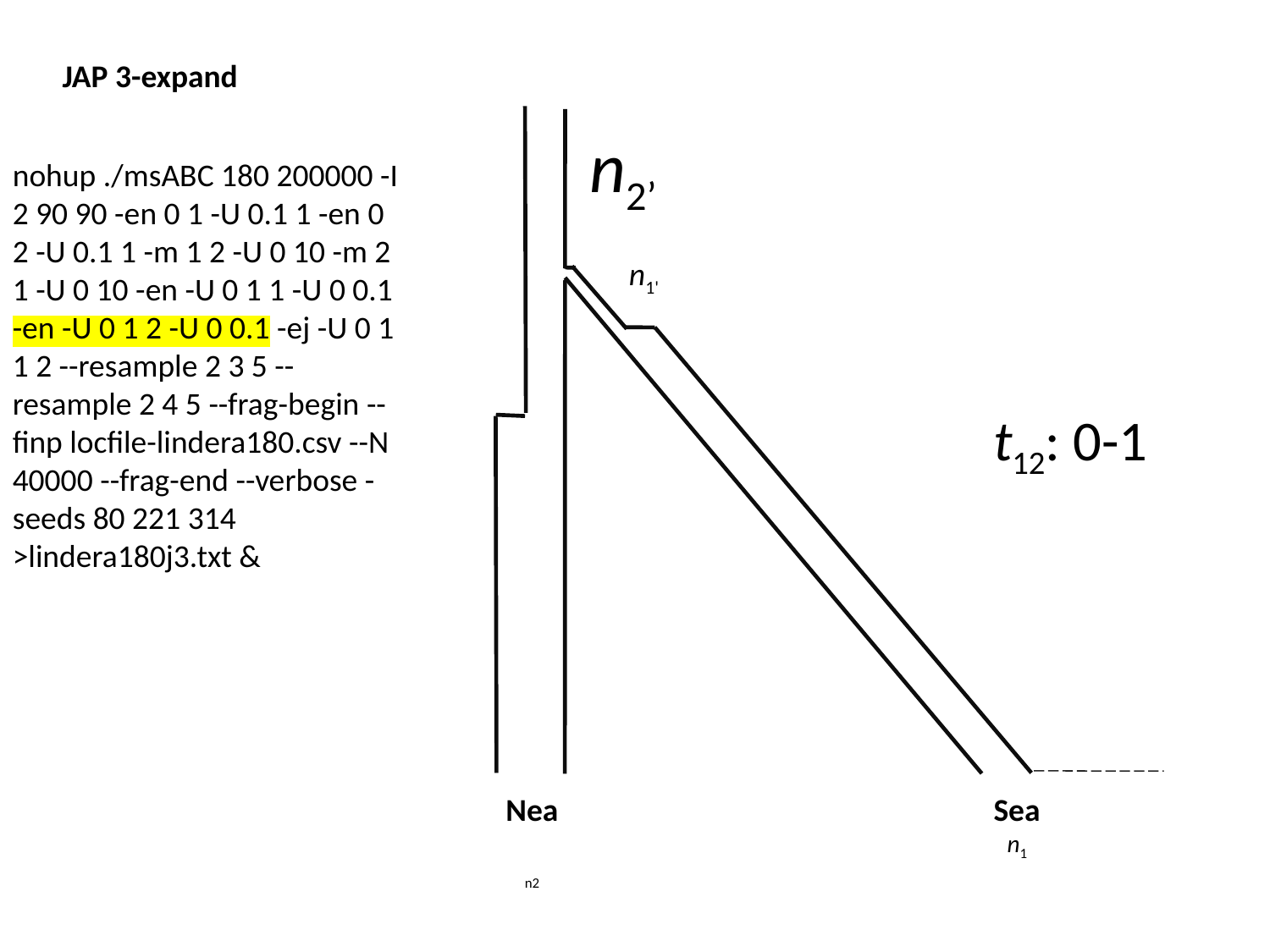

JAP 3-expand
n2’
nohup ./msABC 180 200000 -I 2 90 90 -en 0 1 -U 0.1 1 -en 0 2 -U 0.1 1 -m 1 2 -U 0 10 -m 2 1 -U 0 10 -en -U 0 1 1 -U 0 0.1
-en -U 0 1 2 -U 0 0.1 -ej -U 0 1 1 2 --resample 2 3 5 --resample 2 4 5 --frag-begin --finp locfile-lindera180.csv --N 40000 --frag-end --verbose -seeds 80 221 314 >lindera180j3.txt &
n1'
t12: 0-1
Nea
n2
Sea
n1

## Slide 5
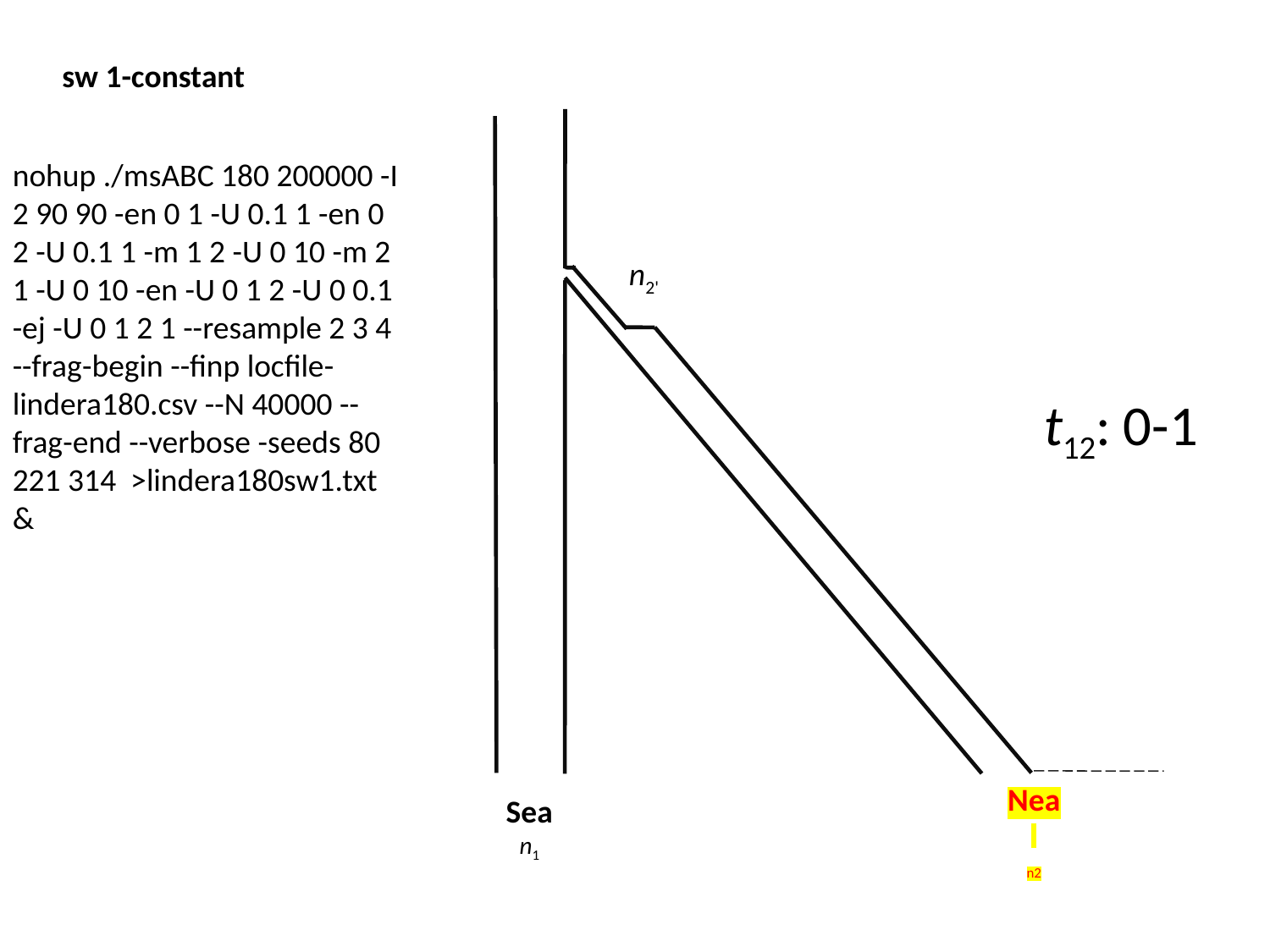

sw 1-constant
nohup ./msABC 180 200000 -I 2 90 90 -en 0 1 -U 0.1 1 -en 0 2 -U 0.1 1 -m 1 2 -U 0 10 -m 2 1 -U 0 10 -en -U 0 1 2 -U 0 0.1 -ej -U 0 1 2 1 --resample 2 3 4 --frag-begin --finp locfile-lindera180.csv --N 40000 --frag-end --verbose -seeds 80 221 314 >lindera180sw1.txt &
n2'
t12: 0-1
Nea
n2
Sea
n1

## Slide 6
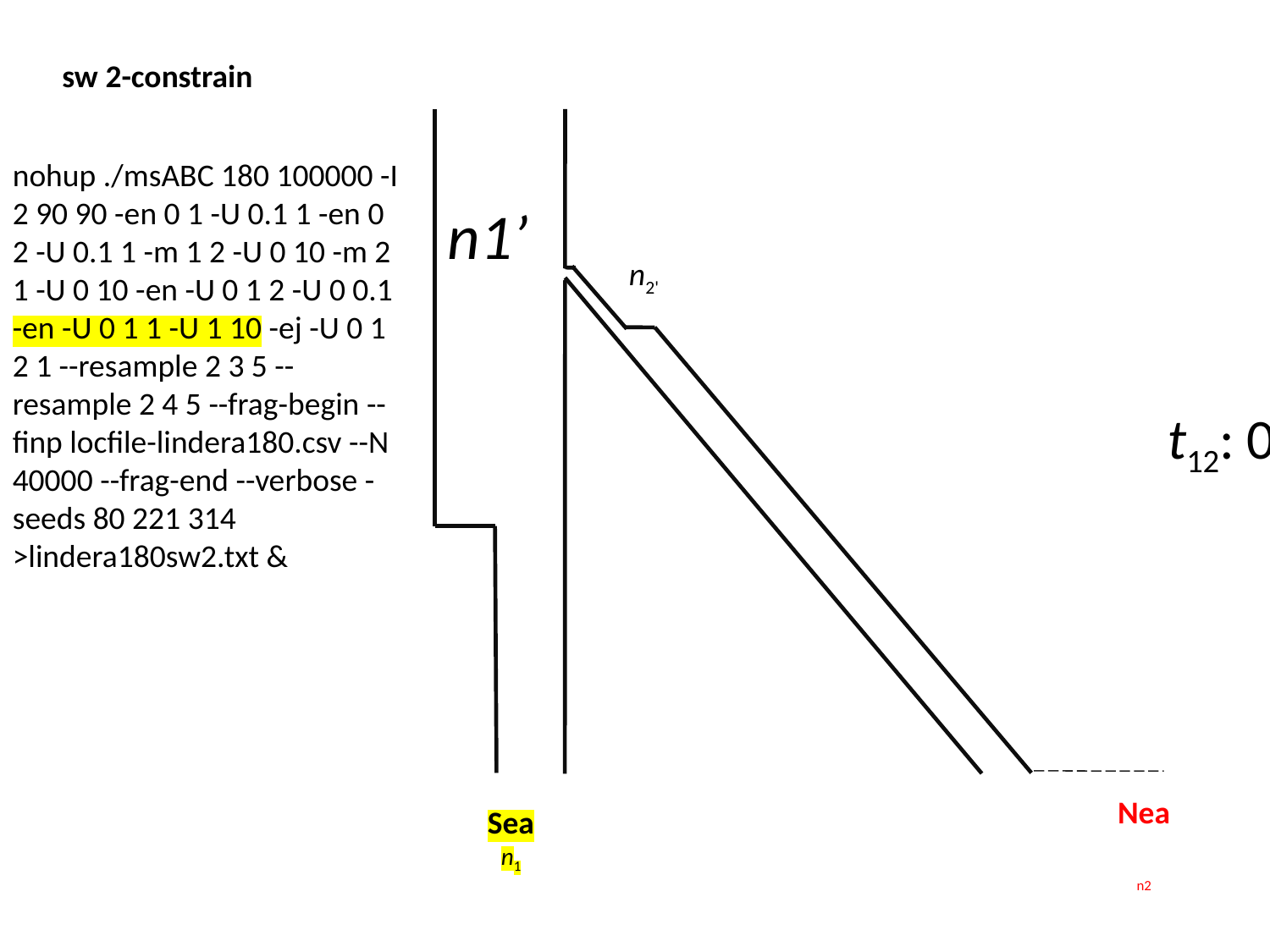

sw 2-constrain
nohup ./msABC 180 100000 -I 2 90 90 -en 0 1 -U 0.1 1 -en 0 2 -U 0.1 1 -m 1 2 -U 0 10 -m 2 1 -U 0 10 -en -U 0 1 2 -U 0 0.1
-en -U 0 1 1 -U 1 10 -ej -U 0 1 2 1 --resample 2 3 5 --resample 2 4 5 --frag-begin --finp locfile-lindera180.csv --N 40000 --frag-end --verbose -seeds 80 221 314 >lindera180sw2.txt &
n1’
n2'
t12: 0-1
Nea
n2
Sea
n1

## Slide 7
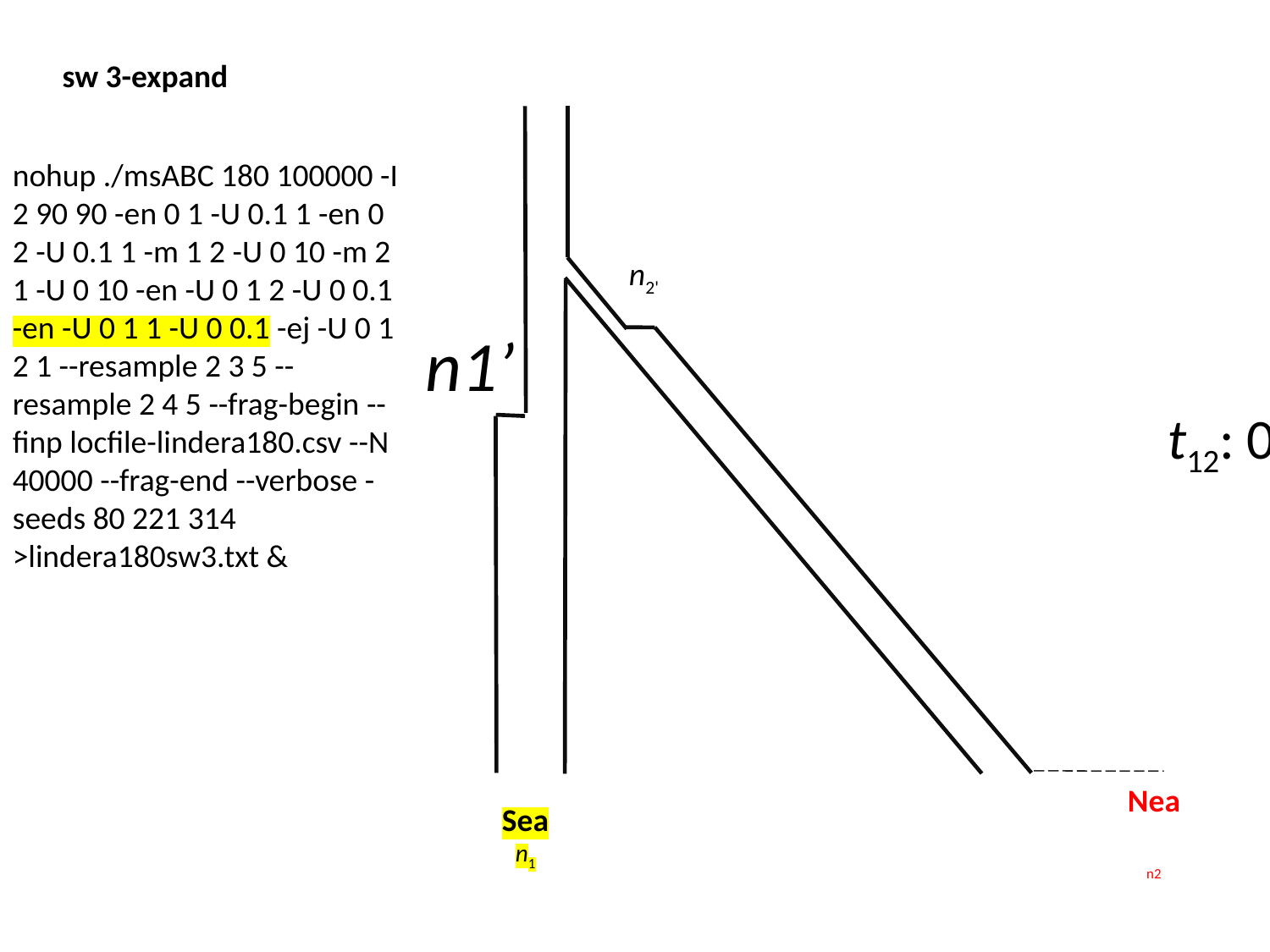

sw 3-expand
nohup ./msABC 180 100000 -I 2 90 90 -en 0 1 -U 0.1 1 -en 0 2 -U 0.1 1 -m 1 2 -U 0 10 -m 2 1 -U 0 10 -en -U 0 1 2 -U 0 0.1
-en -U 0 1 1 -U 0 0.1 -ej -U 0 1 2 1 --resample 2 3 5 --resample 2 4 5 --frag-begin --finp locfile-lindera180.csv --N 40000 --frag-end --verbose -seeds 80 221 314 >lindera180sw3.txt &
n2'
n1’
t12: 0-1
Nea
n2
Sea
n1

## Slide 8
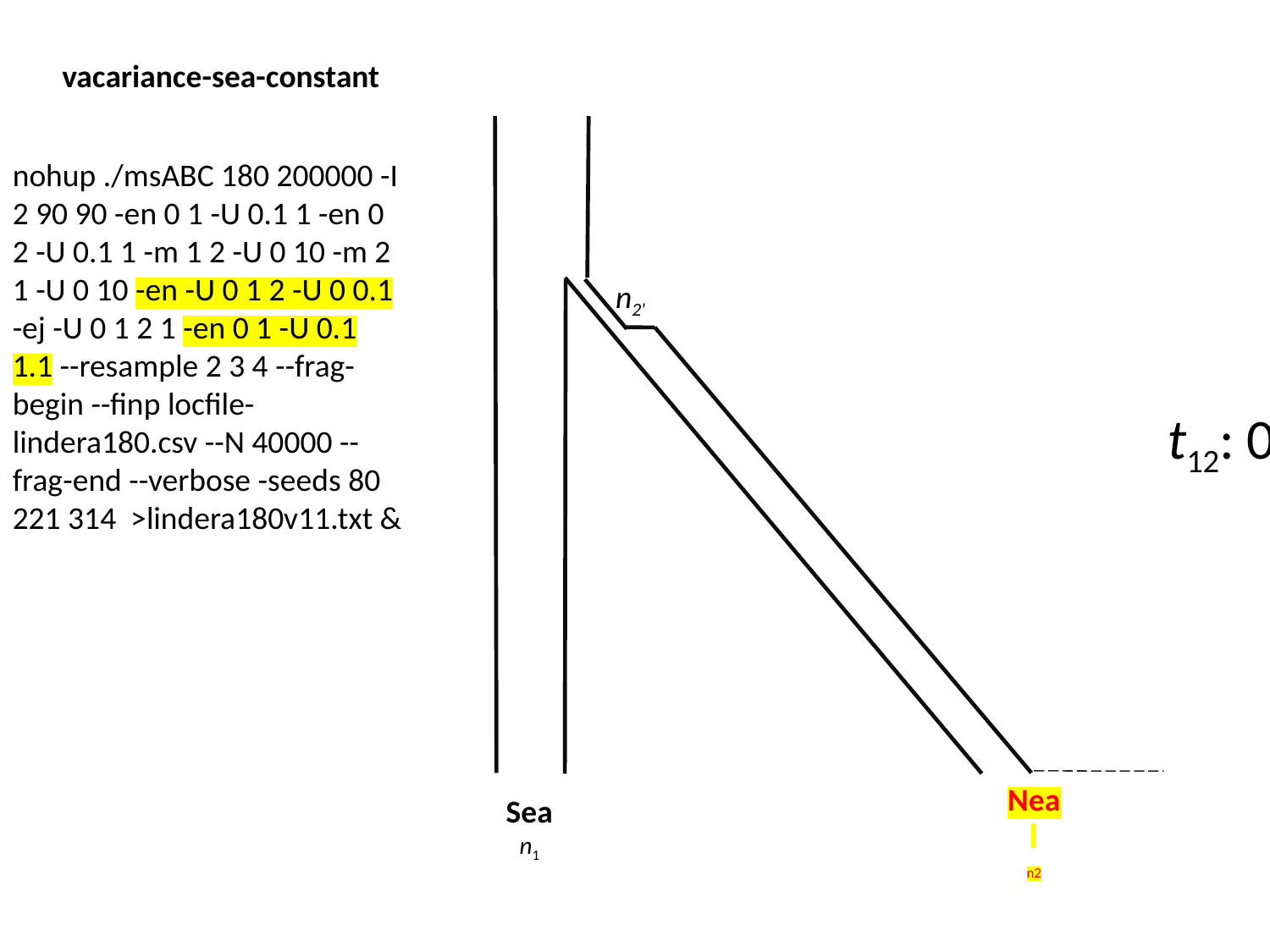

vacariance-sea-constant
nohup ./msABC 180 200000 -I 2 90 90 -en 0 1 -U 0.1 1 -en 0 2 -U 0.1 1 -m 1 2 -U 0 10 -m 2 1 -U 0 10 -en -U 0 1 2 -U 0 0.1 -ej -U 0 1 2 1 -en 0 1 -U 0.1 1.1 --resample 2 3 4 --frag-begin --finp locfile-lindera180.csv --N 40000 --frag-end --verbose -seeds 80 221 314 >lindera180v11.txt &
n2'
t12: 0-1
Nea
n2
Sea
n1

## Slide 9
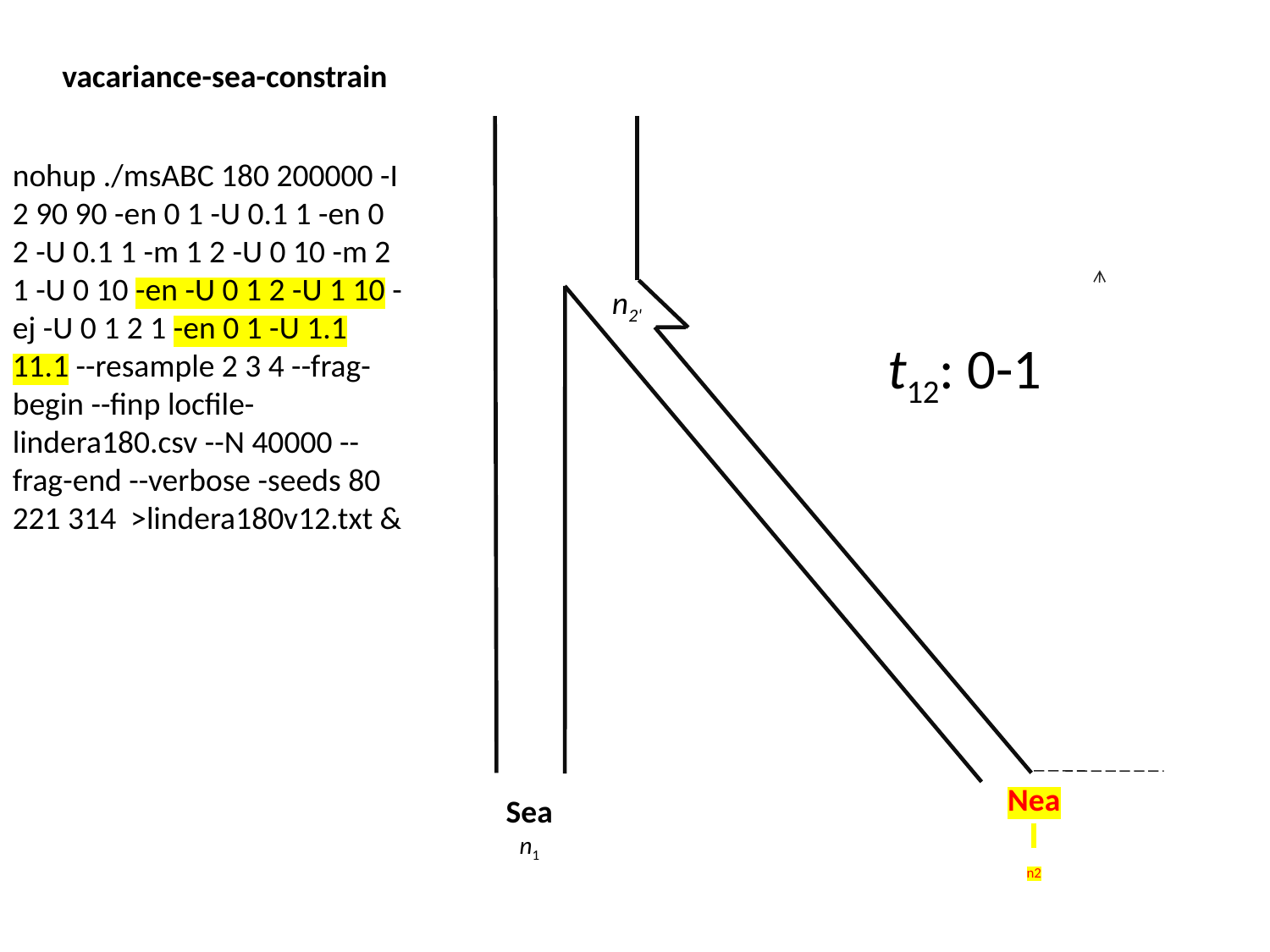

vacariance-sea-constrain
nohup ./msABC 180 200000 -I 2 90 90 -en 0 1 -U 0.1 1 -en 0 2 -U 0.1 1 -m 1 2 -U 0 10 -m 2 1 -U 0 10 -en -U 0 1 2 -U 1 10 -ej -U 0 1 2 1 -en 0 1 -U 1.1 11.1 --resample 2 3 4 --frag-begin --finp locfile-lindera180.csv --N 40000 --frag-end --verbose -seeds 80 221 314 >lindera180v12.txt &
n2'
t12: 0-1
Nea
n2
Sea
n1

## Slide 10
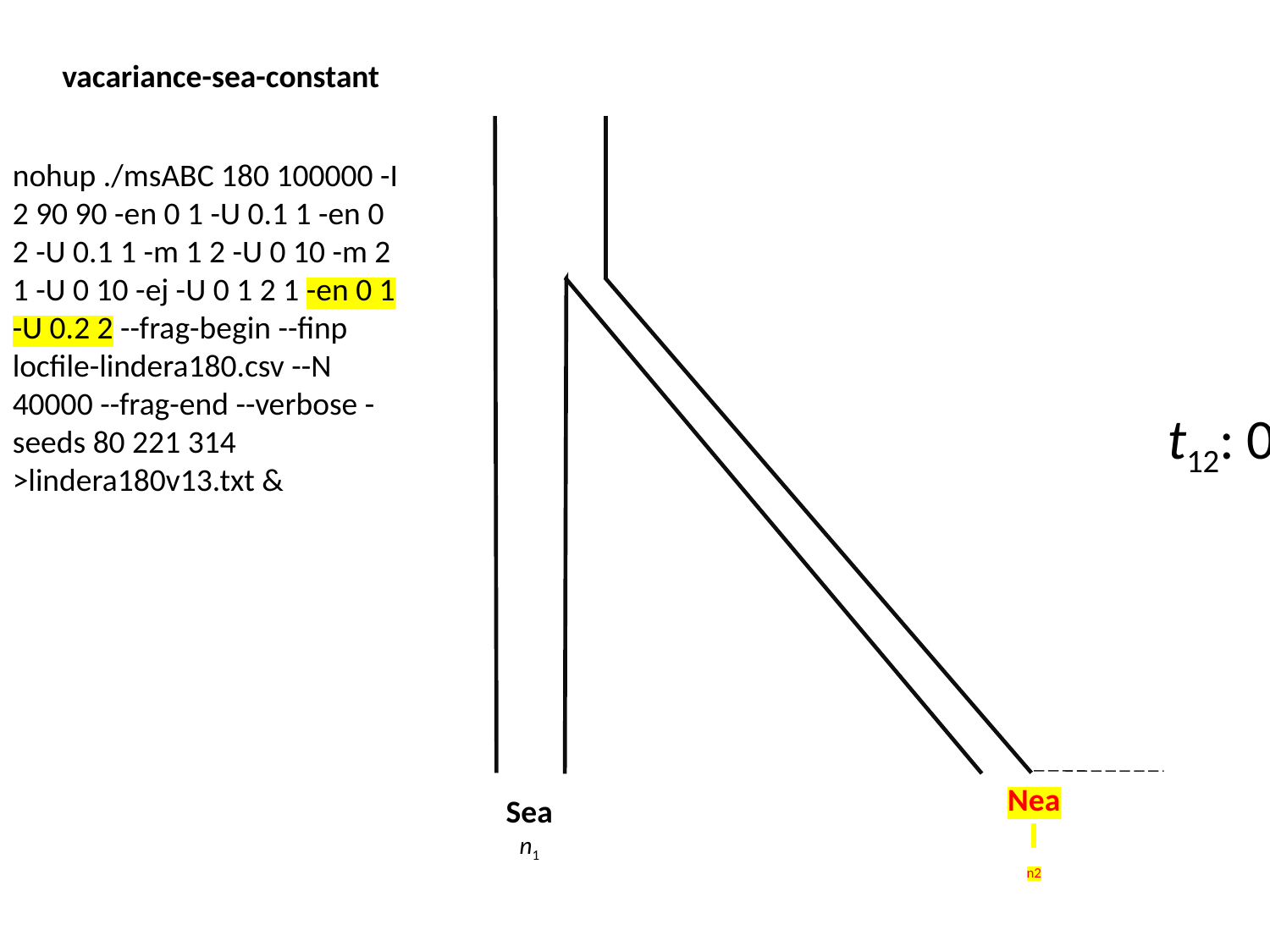

vacariance-sea-constant
nohup ./msABC 180 100000 -I 2 90 90 -en 0 1 -U 0.1 1 -en 0 2 -U 0.1 1 -m 1 2 -U 0 10 -m 2 1 -U 0 10 -ej -U 0 1 2 1 -en 0 1 -U 0.2 2 --frag-begin --finp locfile-lindera180.csv --N 40000 --frag-end --verbose -seeds 80 221 314 >lindera180v13.txt &
t12: 0-1
Nea
n2
Sea
n1

## Slide 11
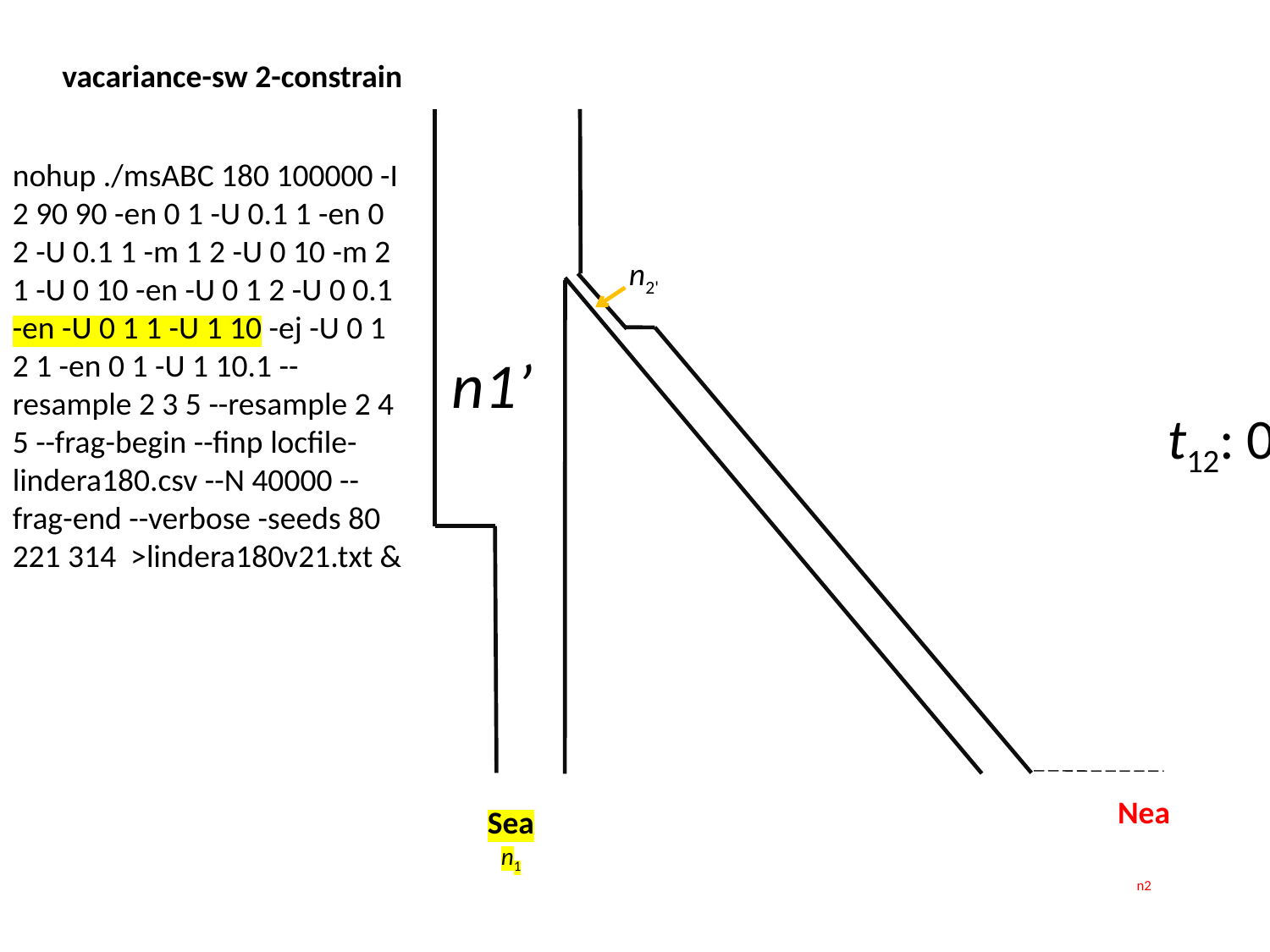

vacariance-sw 2-constrain
nohup ./msABC 180 100000 -I 2 90 90 -en 0 1 -U 0.1 1 -en 0 2 -U 0.1 1 -m 1 2 -U 0 10 -m 2 1 -U 0 10 -en -U 0 1 2 -U 0 0.1
-en -U 0 1 1 -U 1 10 -ej -U 0 1 2 1 -en 0 1 -U 1 10.1 --resample 2 3 5 --resample 2 4 5 --frag-begin --finp locfile-lindera180.csv --N 40000 --frag-end --verbose -seeds 80 221 314 >lindera180v21.txt &
n2'
n1’
t12: 0-1
Nea
n2
Sea
n1

## Slide 12
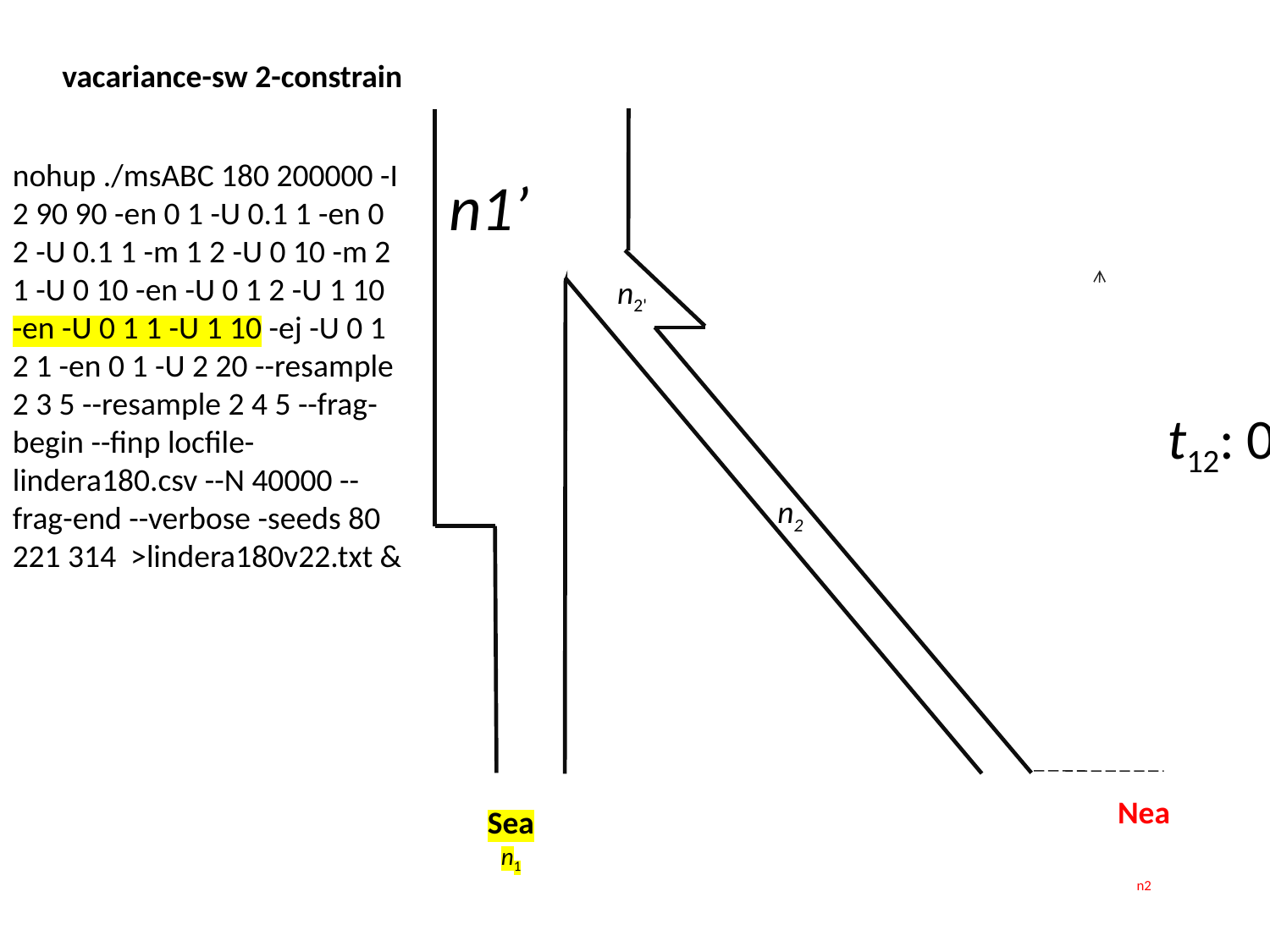

vacariance-sw 2-constrain
nohup ./msABC 180 200000 -I 2 90 90 -en 0 1 -U 0.1 1 -en 0 2 -U 0.1 1 -m 1 2 -U 0 10 -m 2 1 -U 0 10 -en -U 0 1 2 -U 1 10
-en -U 0 1 1 -U 1 10 -ej -U 0 1 2 1 -en 0 1 -U 2 20 --resample 2 3 5 --resample 2 4 5 --frag-begin --finp locfile-lindera180.csv --N 40000 --frag-end --verbose -seeds 80 221 314 >lindera180v22.txt &
n1’
n2'
t12: 0-1
n2
Nea
n2
Sea
n1

## Slide 13
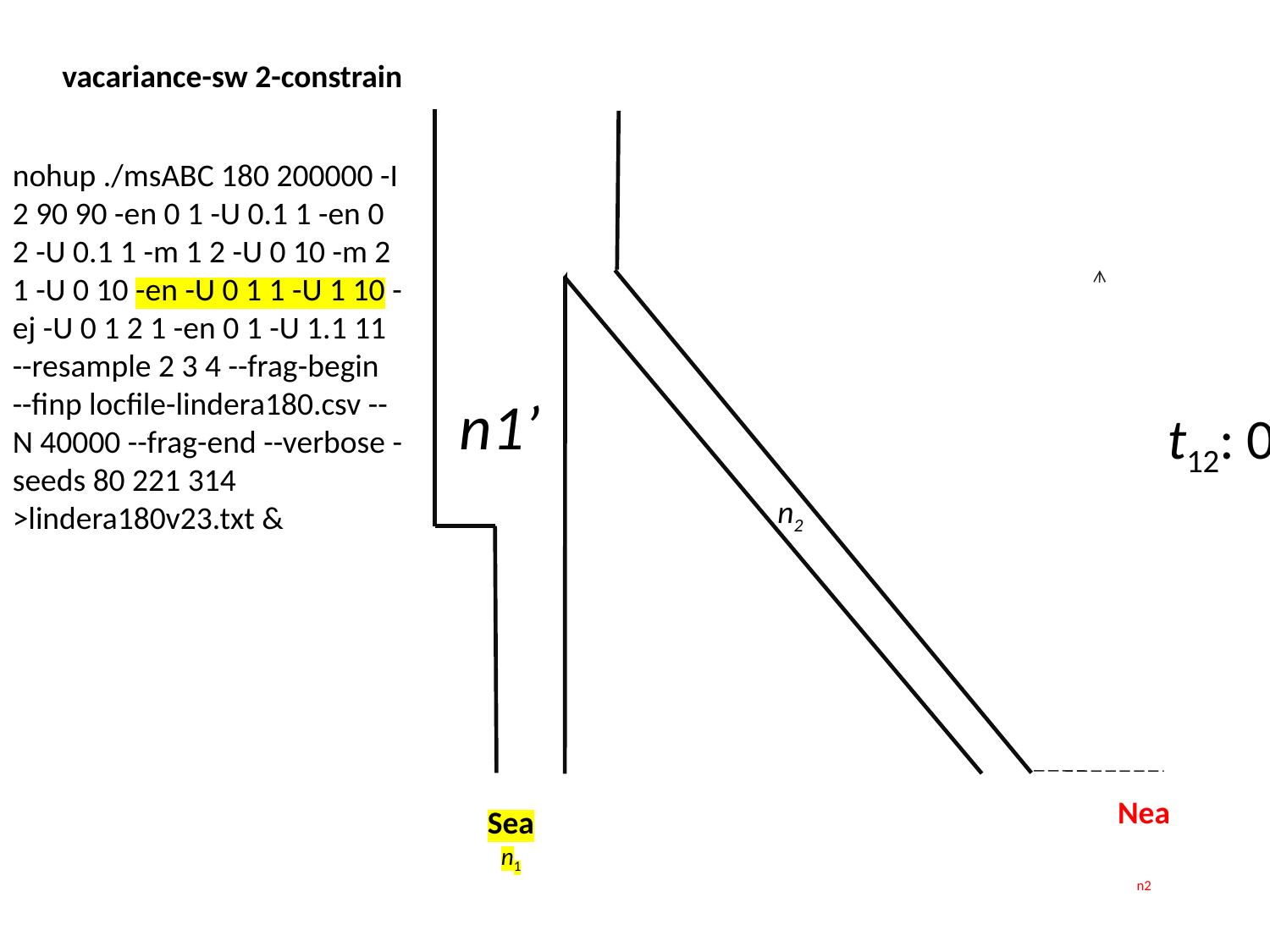

vacariance-sw 2-constrain
nohup ./msABC 180 200000 -I 2 90 90 -en 0 1 -U 0.1 1 -en 0 2 -U 0.1 1 -m 1 2 -U 0 10 -m 2 1 -U 0 10 -en -U 0 1 1 -U 1 10 -ej -U 0 1 2 1 -en 0 1 -U 1.1 11 --resample 2 3 4 --frag-begin --finp locfile-lindera180.csv --N 40000 --frag-end --verbose -seeds 80 221 314 >lindera180v23.txt &
n1’
t12: 0-1
n2
Nea
n2
Sea
n1

## Slide 14
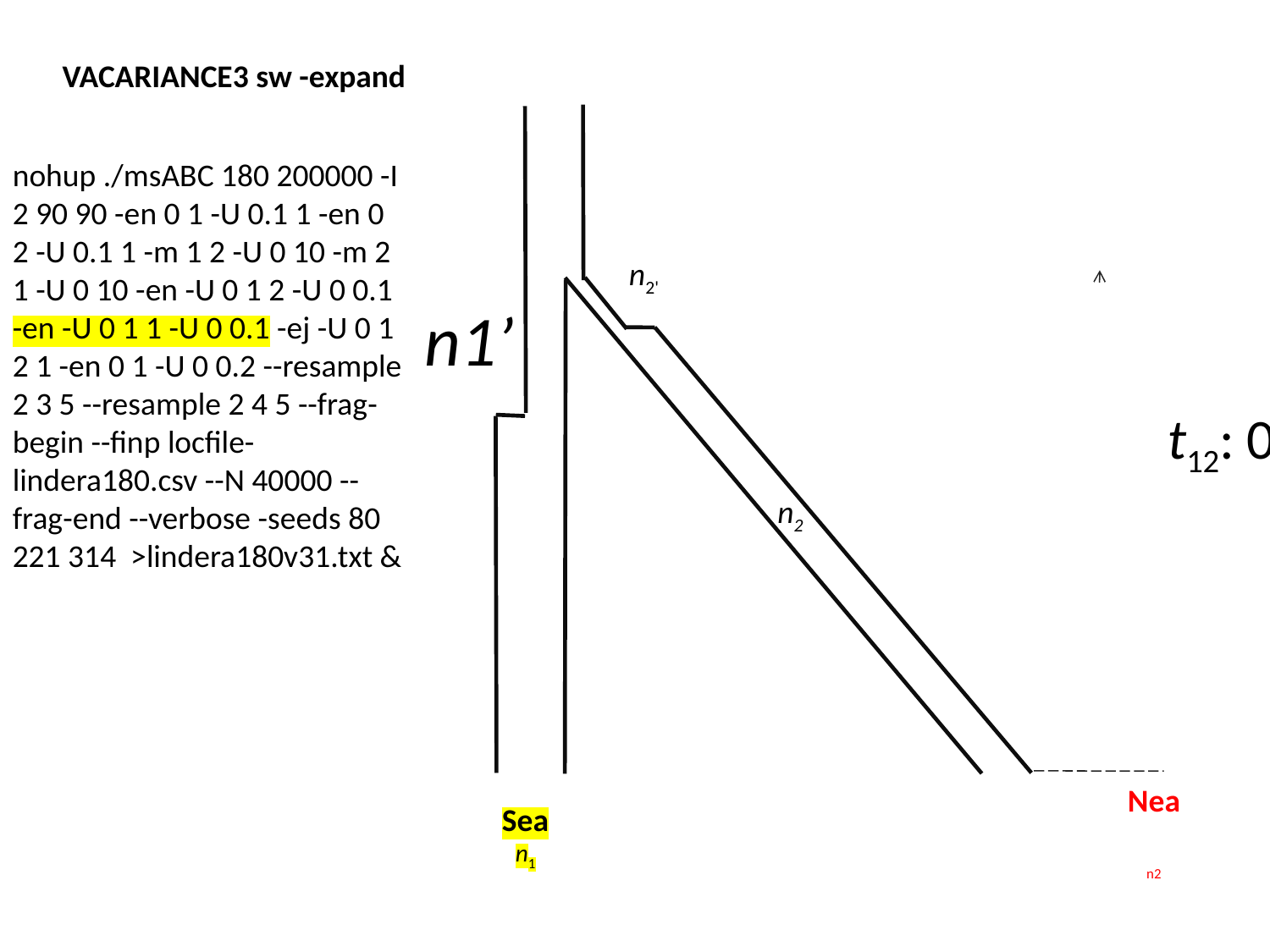

VACARIANCE3 sw -expand
nohup ./msABC 180 200000 -I 2 90 90 -en 0 1 -U 0.1 1 -en 0 2 -U 0.1 1 -m 1 2 -U 0 10 -m 2 1 -U 0 10 -en -U 0 1 2 -U 0 0.1
-en -U 0 1 1 -U 0 0.1 -ej -U 0 1 2 1 -en 0 1 -U 0 0.2 --resample 2 3 5 --resample 2 4 5 --frag-begin --finp locfile-lindera180.csv --N 40000 --frag-end --verbose -seeds 80 221 314 >lindera180v31.txt &
n2'
n1’
t12: 0-1
n2
Nea
n2
Sea
n1

## Slide 15
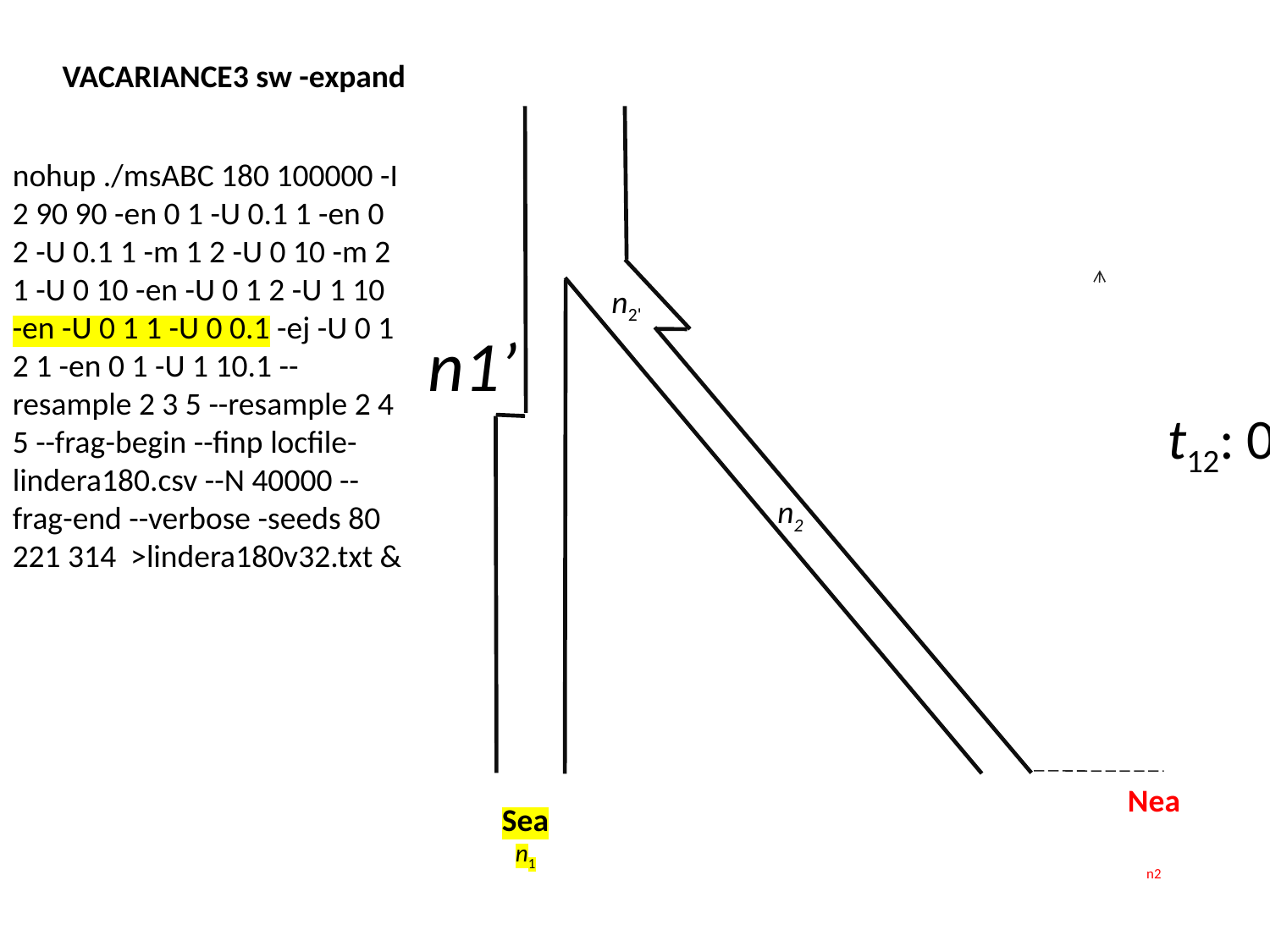

VACARIANCE3 sw -expand
nohup ./msABC 180 100000 -I 2 90 90 -en 0 1 -U 0.1 1 -en 0 2 -U 0.1 1 -m 1 2 -U 0 10 -m 2 1 -U 0 10 -en -U 0 1 2 -U 1 10
-en -U 0 1 1 -U 0 0.1 -ej -U 0 1 2 1 -en 0 1 -U 1 10.1 --resample 2 3 5 --resample 2 4 5 --frag-begin --finp locfile-lindera180.csv --N 40000 --frag-end --verbose -seeds 80 221 314 >lindera180v32.txt &
n2'
n1’
t12: 0-1
n2
Nea
n2
Sea
n1

## Slide 16
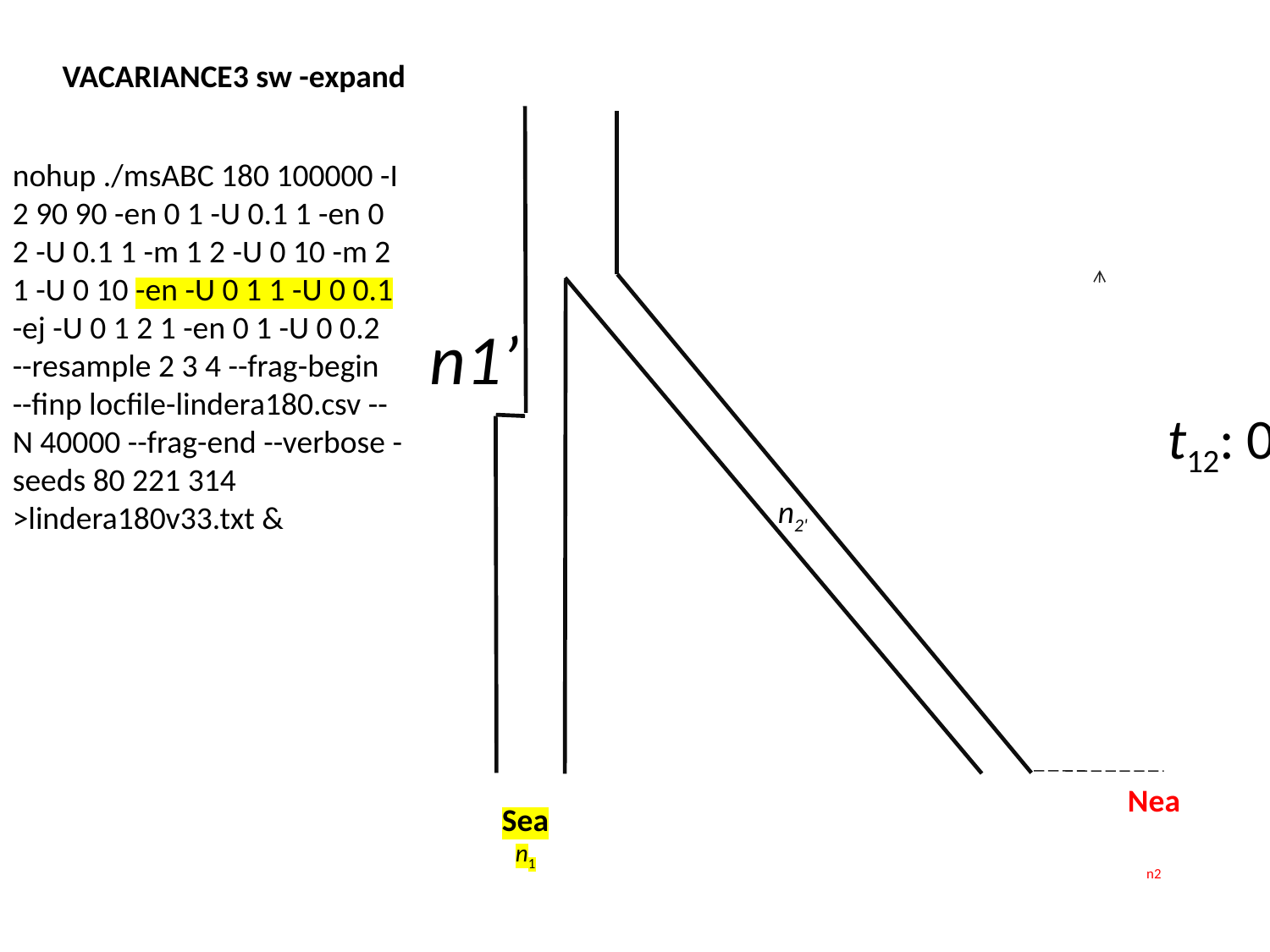

VACARIANCE3 sw -expand
nohup ./msABC 180 100000 -I 2 90 90 -en 0 1 -U 0.1 1 -en 0 2 -U 0.1 1 -m 1 2 -U 0 10 -m 2 1 -U 0 10 -en -U 0 1 1 -U 0 0.1 -ej -U 0 1 2 1 -en 0 1 -U 0 0.2 --resample 2 3 4 --frag-begin --finp locfile-lindera180.csv --N 40000 --frag-end --verbose -seeds 80 221 314 >lindera180v33.txt &
n1’
t12: 0-1
n2'
Nea
n2
Sea
n1
