# Supplementary material for: Complex evolution of East Asian Tertiary relict species revealed by the phylogeography of Lindera obtusiloba
Source: BMC Plant Biol. 2025 Dec 9;26:74. doi: 10.1186/s12870-025-07827-6 (PMC12801852; doi:10.1186/s12870-025-07827-6)
Supplement: Supplementary file 2 — Supplementary Material 2. Table S1 Genetic diversity and neutrality test in 27 nuclear low-copy genes of Lindera obtusiloba. Figure S1 The five major floristic divisions, Eastern Himalayan Province (A), Sikang–Yunnan Province (B), Central Chinese Province (C), North Chinese Province (D) and Japanese–Korean Province (E), in East Asia according to Wu and Wu [1]. Figure S2 Delta-K and LnP(D) values from the STRUCTURE analysis on 24 Lindera obtusiloba populations using 27 nuclear low-copy genes with predefined group number K = 1–20. Standard deviations of LnP(D) obtained from 10 independent runs for each group number are also shown. Figure S3 IMa2 analysis results of Lindera obtusiloba populations using 27 low-copy nuclear genes. Posterior probability distributions of divergence time (t) between NEA and SEA populations (a) and effective population size of NEA, SEA and Ancestral populations (b) are illustrated. Figure S4 Posterior probability distributions of estimated demographic parameters in SEA origin (expansion) scenario. 1 and 2 represent populations in SEA and NEA, respectively. Figure S5 Posterior probability distributions of estimated demographic parameters in NEA origin (expansion) scenario. 1 and 2 represent populations in SEA and NEA, respectively. Table S2 Voucher numbers for 24 sampled populations of Lindera obtusiloba. Table S3 Bio-climatic variables, standardized loading for the two first axes of the principal component analysis (PCA) (present climate) and result of isolation by environment (IBE) analysis. Figure S6 Ancestral area reconstructions derived by DEC+j model usging BioGeoBEARS base on the BEAST-derived chronograms of 24 populations of Lindera obtusiloba with outgroup L. erythrocarpa (ZHGS) using 15 low-copy nuclear gene loci (the remaining 12 loci were not successfully sequenced in L. erythrocarpa) based on the estimated substitution rate, mean: 4.58 × 10^-9^ (95% highest-probability-density interval, HPD, : 1.66–12.69 × 10-9). Five geographic re [file 12870_2025_7827_MOESM2_ESM.docx]

**Supporting Information**

Complex evolution of East Asian Tertiary relict species revealed by the phylogeography of *Lindera obtusiloba*

Jun-Wei Ye, Rui Yang, Lei Bao, Meng-Jing Dai, Hong-Fang Wang, Jian-Ping Ge

| **Table S1** Genetic diversity and neutrality test in 27 nuclear low-copy genes of *Lindera obtusiloba.* | | | | | | | | | | | | |
| --- | --- | --- | --- | --- | --- | --- | --- | --- | --- | --- | --- | --- |
| Locus | Length | n | V | S | P | Hap | Hd | π (×10^-3^) | Theta (×10^-3^) | Ewens -Watterson test | Fu's Fs | Tajima's D |
| 2AP | 810 | 164 | 41 | 11 | 30 | 31 | 0.88 | 8.04 | 8.92 | 0.916 | -4.197 | -0.295 |
| 2DA | 581 | 166 | 23 | 4 | 19 | 18 | 0.88 | 8.19 | 6.96 | 0.211 | -0.028 | 0.493 |
| ACY | 944 | 170 | 71 | 14 | 57 | 49 | 0.97 | 8.53 | 13.17 | 0.238 | -15.415^*^ | -1.087 |
| BAE | 629 | 170 | 27 | 7 | 20 | 26 | 0.83 | 6.59 | 7.52 | 0.954^*^ | -5.472 | -0.35 |
| COD1 | 154 | 174 | 10 | 2 | 8 | 13 | 0.73 | 8.55 | 11.33 | 0.744 | -4.379 | -0.582 |
| FASP | 626 | 174 | 23 | 7 | 16 | 21 | 0.79 | 6.18 | 6.41 | 0.921 | -2.688 | -0.099 |
| GPN | 516 | 174 | 17 | 9 | 8 | 15 | 0.72 | 4.35 | 5.75 | 0.877 | -2.592 | -0.643 |
| HET | 612 | 174 | 22 | 4 | 18 | 15 | 0.82 | 7.69 | 6.27 | 0.458 | 1.448 | 0.623 |
| HIST | 228 | 168 | 11 | 3 | 8 | 12 | 0.78 | 6.57 | 8.47 | 0.412 | -2.8 | -0.546 |
| HPT | 389 | 170 | 23 | 10 | 13 | 23 | 0.84 | 4.91 | 10.35 | 0.858 | -12.239^**^ | -1.462 |
| HYPO | 305 | 174 | 16 | 4 | 12 | 15 | 0.72 | 6.65 | 9.15 | 0.881 | -3.222 | -0.714 |
| INTE | 324 | 174 | 14 | 3 | 11 | 13 | 0.70 | 8.35 | 7.54 | 0.83 | -0.427 | 0.275 |
| ISOM | 341 | 174 | 16 | 5 | 11 | 13 | 0.75 | 5.39 | 8.21 | 0.722 | -2.38 | -0.897 |
| LEP2 | 391 | 162 | 20 | 10 | 10 | 18 | 0.68 | 5.16 | 9.03 | 0.983^*^ | -6.007^*^ | -1.17 |
| LG3 | 472 | 172 | 16 | 4 | 12 | 16 | 0.84 | 3.55 | 5.92 | 0.46 | -5.503* | -1.049 |
| LPD | 187 | 176 | 3 | 0 | 3 | 4 | 0.56 | 3.37 | 2.79 | 0.242 | 0.512 | 0.337 |
| MALA | 328 | 174 | 11 | 0 | 11 | 11 | 0.70 | 5.85 | 5.85 | 0.673 | -0.867 | -0.001 |
| MPD | 622 | 172 | 20 | 2 | 18 | 16 | 0.82 | 6.27 | 5.62 | 0.582 | -0.119 | 0.314 |
| PENT | 456 | 172 | 11 | 3 | 8 | 12 | 0.57 | 1.60 | 4.22 | 0.952^*^ | -0.749^*^ | -1.507 |
| PORI | 418 | 174 | 12 | 4 | 8 | 10 | 0.67 | 6.05 | 5.02 | 0.691 | 0.942 | 0.506 |
| PRUP | 586 | 176 | 22 | 4 | 18 | 21 | 0.85 | 4.75 | 6.54 | 0.659 | -0.542 | -0.75 |
| SPT2 | 497 | 172 | 22 | 7 | 15 | 20 | 0.81 | 4.91 | 4.94 | 0.863 | -5.9^*^ | -1.008 |
| STOP | 600 | 178 | 30 | 9 | 21 | 23 | 0.80 | 8.56 | 8.69 | 0.953^*^ | -1.67 | -0.042 |
| STP | 386 | 176 | 9 | 2 | 7 | 14 | 0.73 | 3.06 | 4.06 | 0.821 | -0.619^*^ | -0.569 |
| TDM | 677 | 172 | 19 | 1 | 18 | 19 | 0.79 | 4.77 | 4.90 | 0.864 | -2.828 | -0.074 |
| TPP | 614 | 174 | 23 | 8 | 15 | 19 | 0.75 | 5.63 | 6.53 | 0.944 | -2.327 | -0.385 |
| VEST | 592 | 174 | 28 | 12 | 16 | 28 | 0.89 | 4.24 | 8.25 | 0.817 | -14.505 | -1.384 |
| V, variable sites; S, singleton variable sites; P, parsimony informative sites; Hap, haplotypes; ^*^, *P* < 0.05, ^**^, *P* < 0.001 | | | | | | | | | | | | |


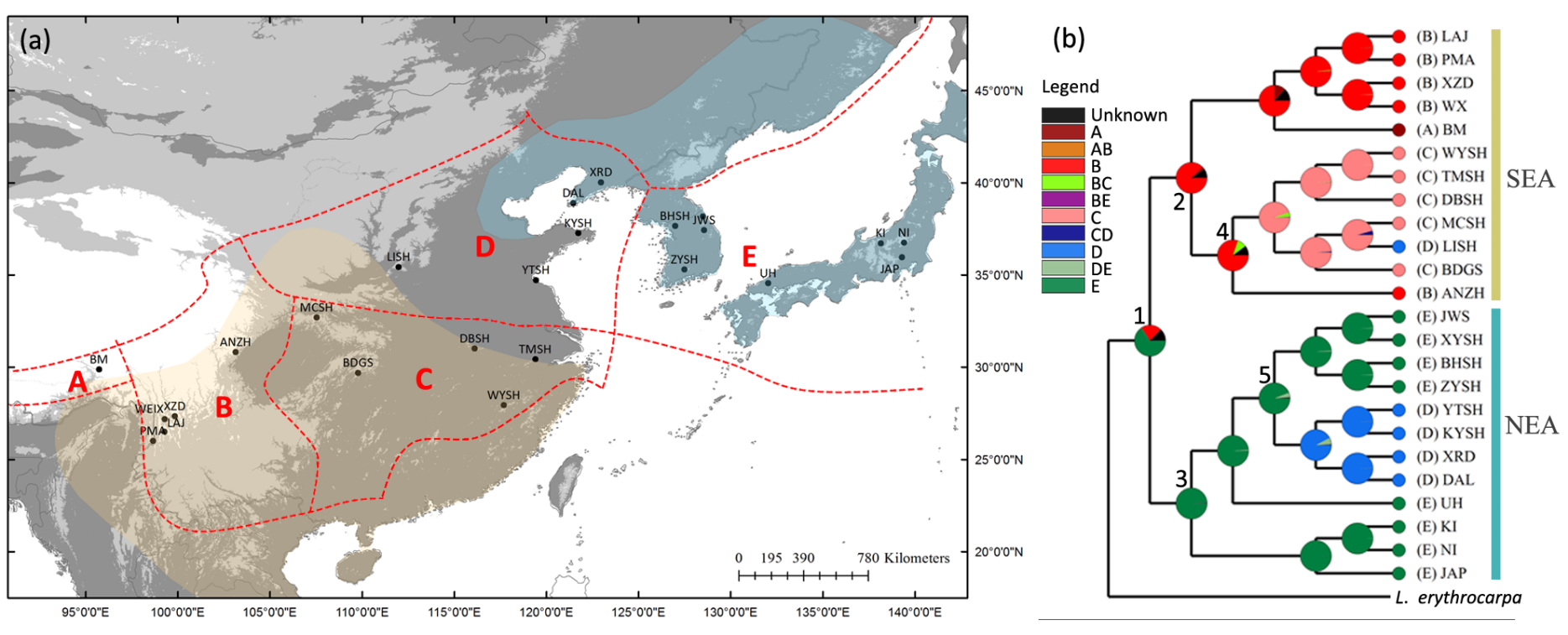


Figure S1 The five major floristic divisions, Eastern Himalayan Province (A), Sikang–Yunnan Province (B), Central Chinese Province (C), North Chinese Province (D) and Japanese–Korean Province (E), in East Asia according to Wu and Wu ([1996](#_ENREF_49)).


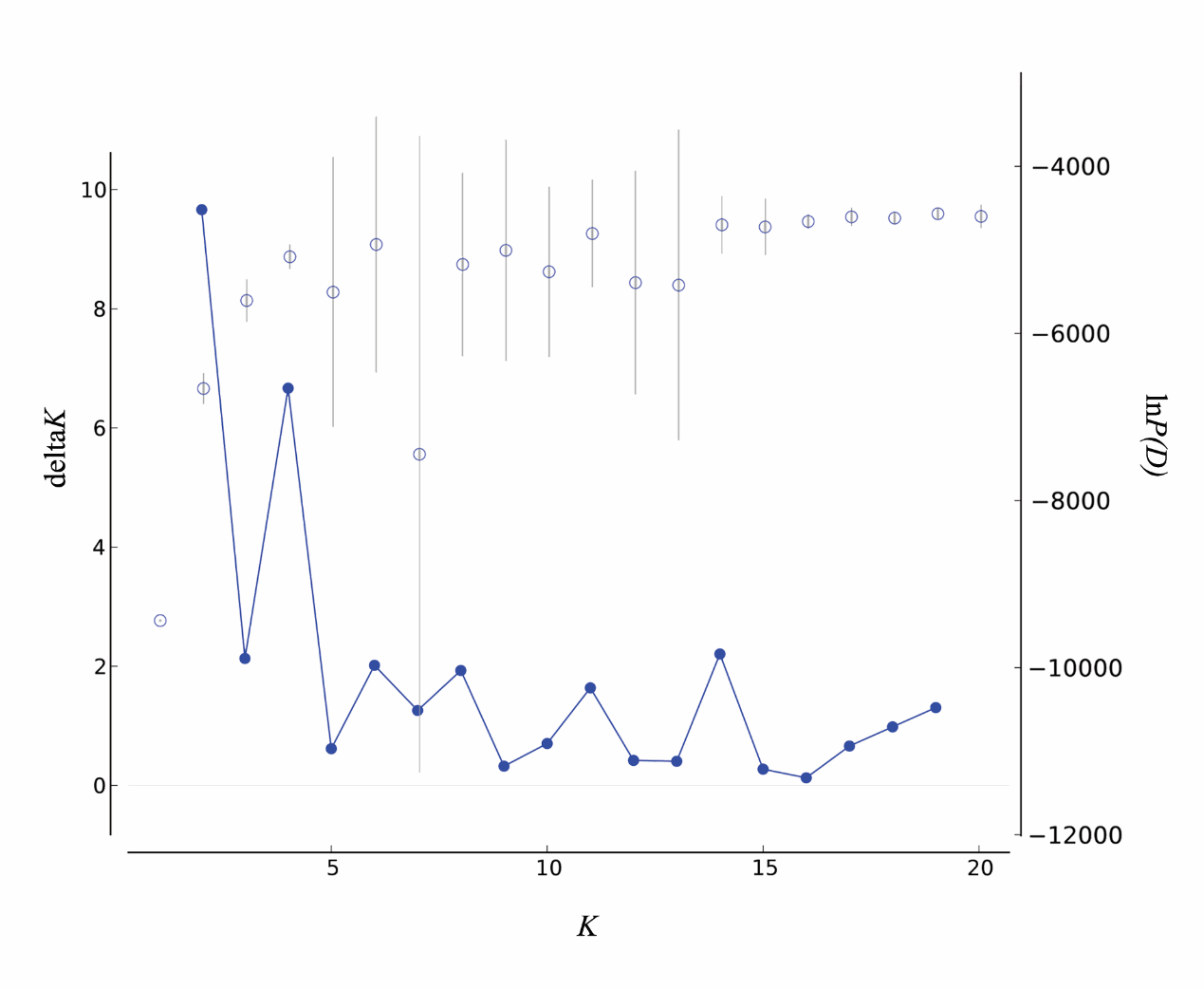


Figure S2 Delta-*K* and Ln*P*(*D*) values from the STUCTURE analysis on 24 *Lindera obtusiloba* populations using 27 nuclear low-copy genes with predefined group number *K* = 1–20. Standard deviations of Ln*P*(*D*) obtained from 10 independent runs for each group number are also shown.


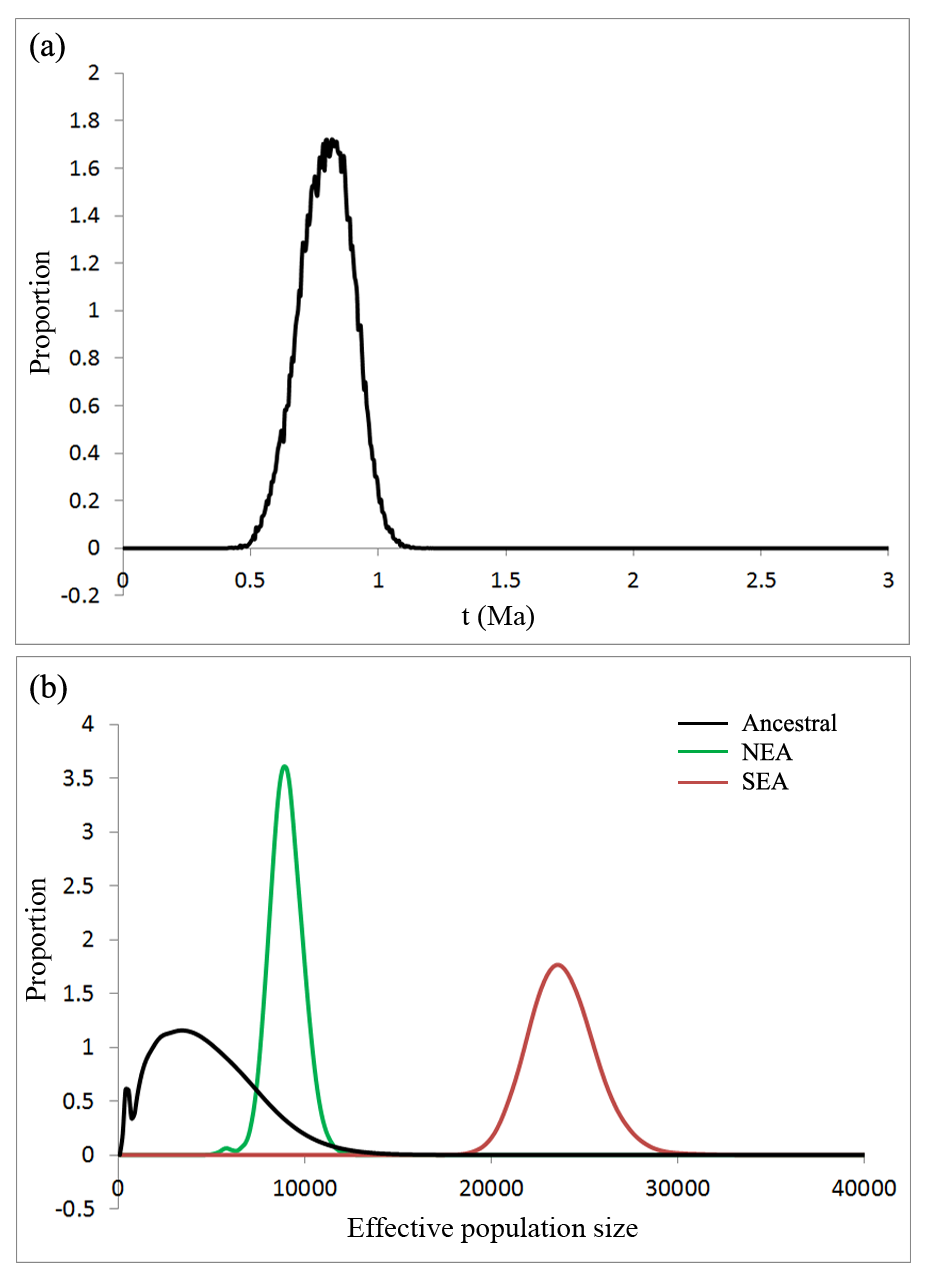


**Figure S3** IMa2 analysis results of *Lindera obtusiloba* populations using 27 low-copy nuclear genes. Posterior probability distributions of divergence time (*t*) between NEA and SEA populations (a) and effective population size of NEA, SEA and Ancestral populations (b) are illustrated.


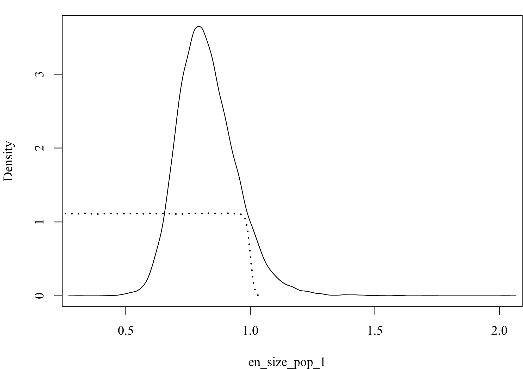

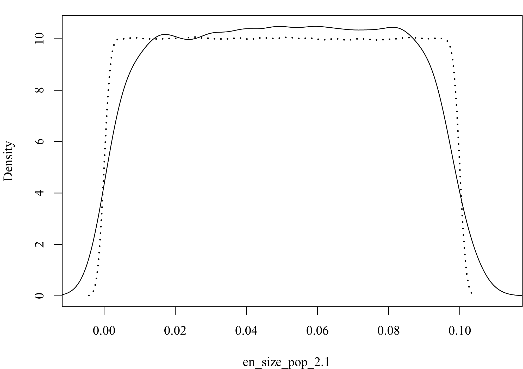

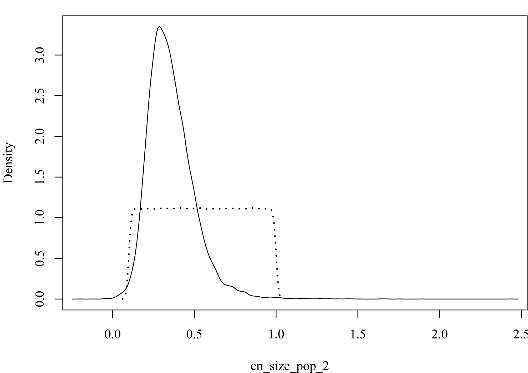

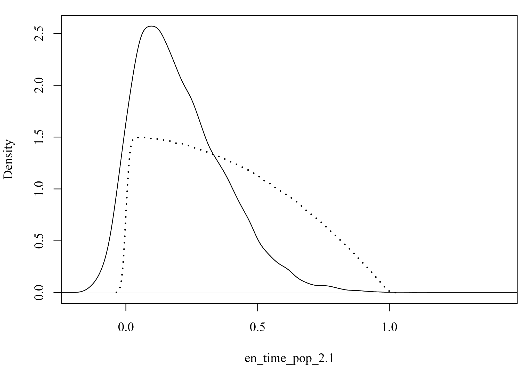

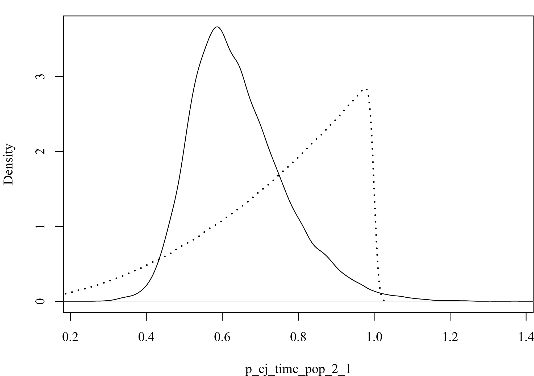

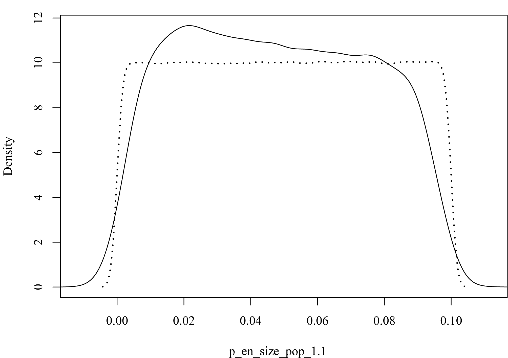

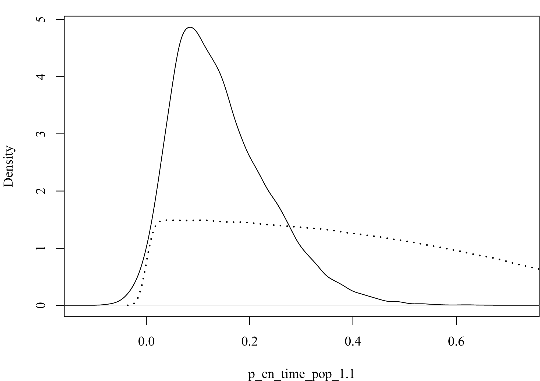

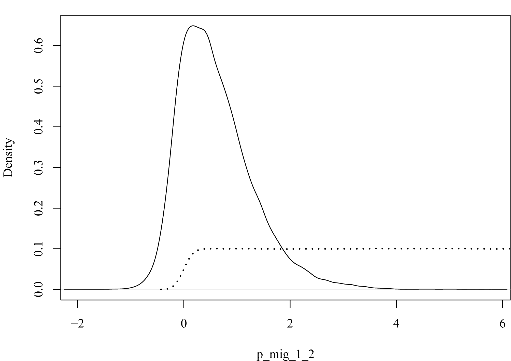

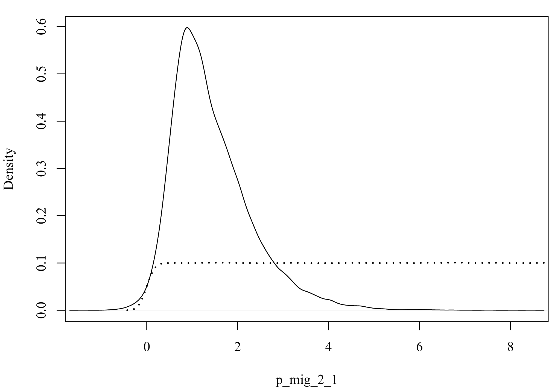


**Figure S4** Posterior probability distributions of estimated demographic parameters in SEA origin (expansion) scenario. 1 and 2 represent populations in SEA and NEA, respectively.


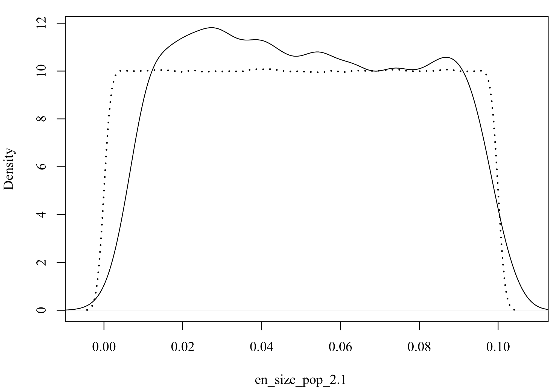

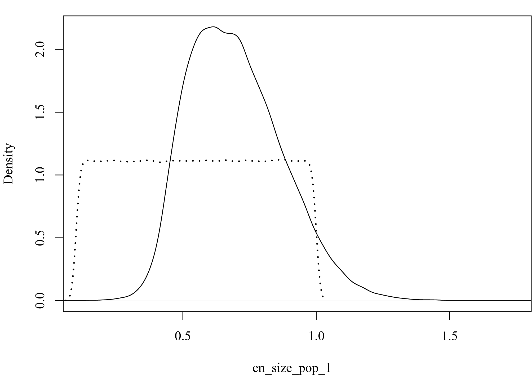

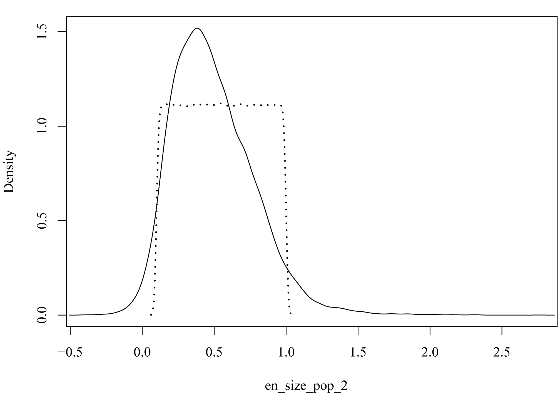

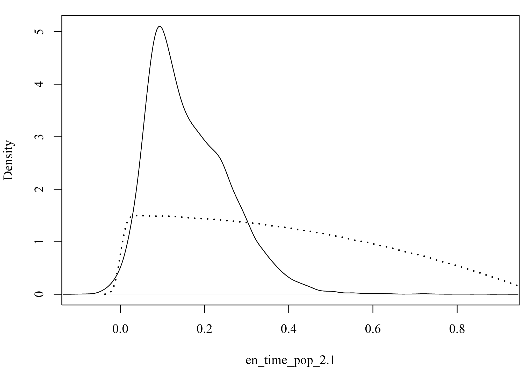

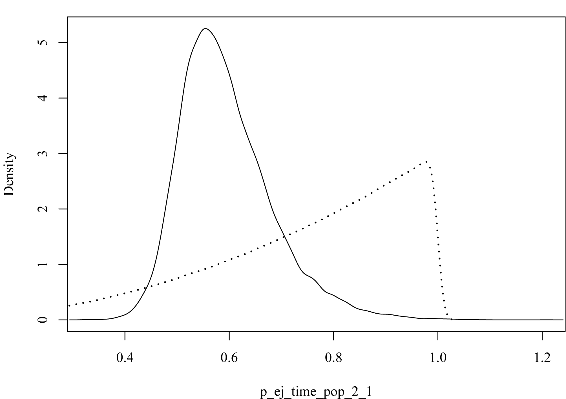

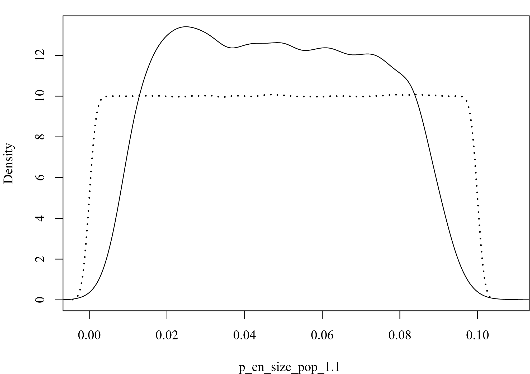

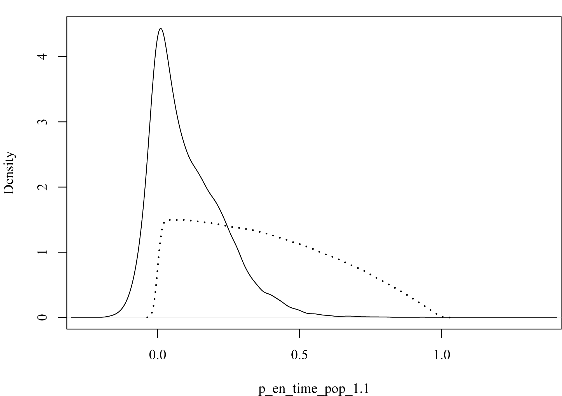

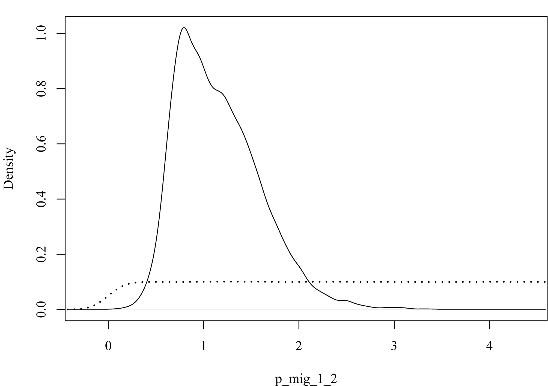

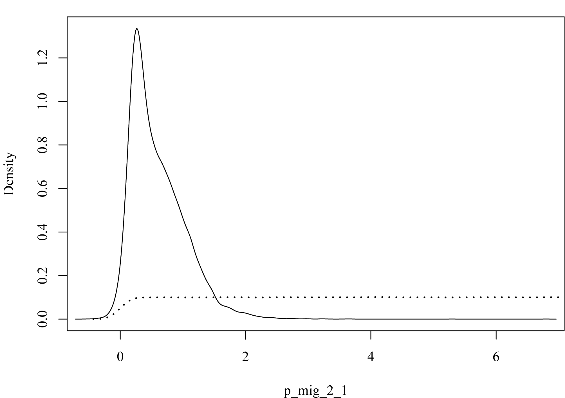


**Figure S5** Posterior probability distributions of estimated demographic parameters in NEA origin (expansion) scenario. 1 and 2 represent populations in SEA and NEA, respectively.

| **Table S2** Voucher numbers for 24 sampled populations of *Lindera obtusiloba*. | | | |
| --- | --- | --- | --- |
| Code | Region/Population | Location | Voucher number |
|  | SEA region |  |  |
| 1 | BM | Bomi, Xizang, China | BNU23259 |
| 2 | PMA | Pianma, Yunnan, China | BNU23240 |
| 3 | LAJ | Lajing, Yunnan, China | BNU23241 |
| 4 | WEIX | Weixi, Yunnan, China | BNU23242 |
| 5 | XZD | Xiaozhongdian, Yunnan, China | BNU23243 |
| 6 | ANZH | Anzihe Nature Reserve, Sichuan, China | BNU23248 |
| 7 | MCSH | Mt. Micang, Shannxi, China | BNU23249 |
| 8 | BDGS | Mt. Badagong, Hunan, China | BNU23250 |
| 9 | LISH | Mt. Li, Shanxi, China | BNU23244 |
| 10 | DBSH | Mt. Daba, Anhui, China | BNU23245 |
| 11 | WYSH | Mt. Wuyi, Jiangxi, China | BNU23246 |
| 12 | TMSH | Mt. Tianmu, Zhejiang, China | BNU23247 |
|  | NEA region |  |  |
| 13 | YTSH | Mt. Yuntai, Jiangsu, China | BNU23236 |
| 14 | DAL | Dalian, Liaoning, China | BNU23237 |
| 15 | KYSH | Mt. Kunyu, Shandong, China | BNU23238 |
| 16 | XRD | Zhuanghe, Liaoning, China | BNU23239 |
| 17 | BHSH | Bukhansan National Park, Seoul City, Korea | BNU23258 |
| 18 | ZYSH | Mt. Jiri, South Gyeongsang Province, Korea | BNU23257 |
| 19 | XYSH | Seoraksan National Park, Gangwon Province, Korea | BNU23256 |
| 20 | JWS | Gariwangsan, Gangwon Province, Korea | BNU23255 |
| 21 | UH | Masuda-shi Shimane-ken, Japan | BNU23251 |
| 22 | KI | Kawakami, Nagano-ken, Japan | BNU23252 |
| 23 | JAP | Tokyo, Japan | BNU23253 |
| 24 | NI | Nikkō-shi, Tochigi-ken, Japan | BNU23254 |

| **Table S3** Bio-climatic variables, standardized loading for the two first axes of the principal component analysis (PCA) (present climate) and result of isolation by environment (IBE) analysis | | | | | |  |
| --- | --- | --- | --- | --- | --- | --- |
| Variable | Description | First axis (PC1) | Second axis (PC2) | *r* | *P* |  |
|  |  |  |  |  |  |  |
| c1 | Component 1 |  |  | 0.02 | 0.36 |  |
| c2 | Component 2 |  |  | **0.22** | **0.01** |  |
| bio1 | Annual mean temperature | 0.27 | 0.07 | 0.09 | 0.11 |  |
| bio2 | Mean diurnal range (mean of monthly (max temp - min temp)) | -0.16 | 0.09 | 0.03 | 0.32 |  |
| bio3 | Isothermality (BIO2/BIO7) (× 100) | -0.15 | **0.35** | 0.11 | 0.10 |  |
| bio4 | Temperature seasonality (SD × 100) | 0.08 | **-0.44** | **0.19** | **0.01** |  |
| bio5 | Max temperature of warmest month | 0.25 | -0.25 | 0.08 | 0.16 |  |
| bio6 | Min temperature of coldest month | 0.22 | 0.27 | **0.11** | **0.06** |  |
| bio7 | Temperature Annual Range (BIO5-BIO6) | 0.03 | **-0.42** | **0.15** | **0.03** |  |
| bio8 | Mean temperature of wettest quarter | 0.21 | -0.24 | 0.09 | 0.13 |  |
| bio9 | Mean temperature of driest quarter | 0.22 | **0.31** | **0.19** | **0.01** |  |
| bio10 | Mean temperature of warmest quarter | 0.26 | -0.23 | 0.09 | 0.12 |  |
| bio11 | Mean temperature of coldest quarter | 0.17 | **0.33** | 0.12 | 0.06 |  |
| bio12 | Annual precipitation | **0.31** | 0.09 | -0.08 | 0.14 |  |
| bio13 | Precipitation of wettest month | 0.20 | 0.06 | **-0.11** | **0.05** |  |
| bio14 | Precipitation of driest month | **0.30** | -0.04 | **-0.27** | **0.00** |  |
| bio15 | Precipitation seasonality (coefficient of variation) | -0.28 | 0.00 | **-0.11** | **0.03** |  |
| bio16 | Precipitation of wettest quarter | 0.22 | 0.14 | -0.08 | 0.13 |  |
| bio17 | Precipitation of driest quarter | **0.31** | -0.02 | **-0.21** | **0.00** |  |
| bio18 | Precipitation of warmest quarter | 0.20 | 0.13 | **-0.13** | **0.02** |  |
| bio19 | Precipitation of coldest quarter | **0.31** | -0.01 | **-0.16** | **0.00** |  |

**
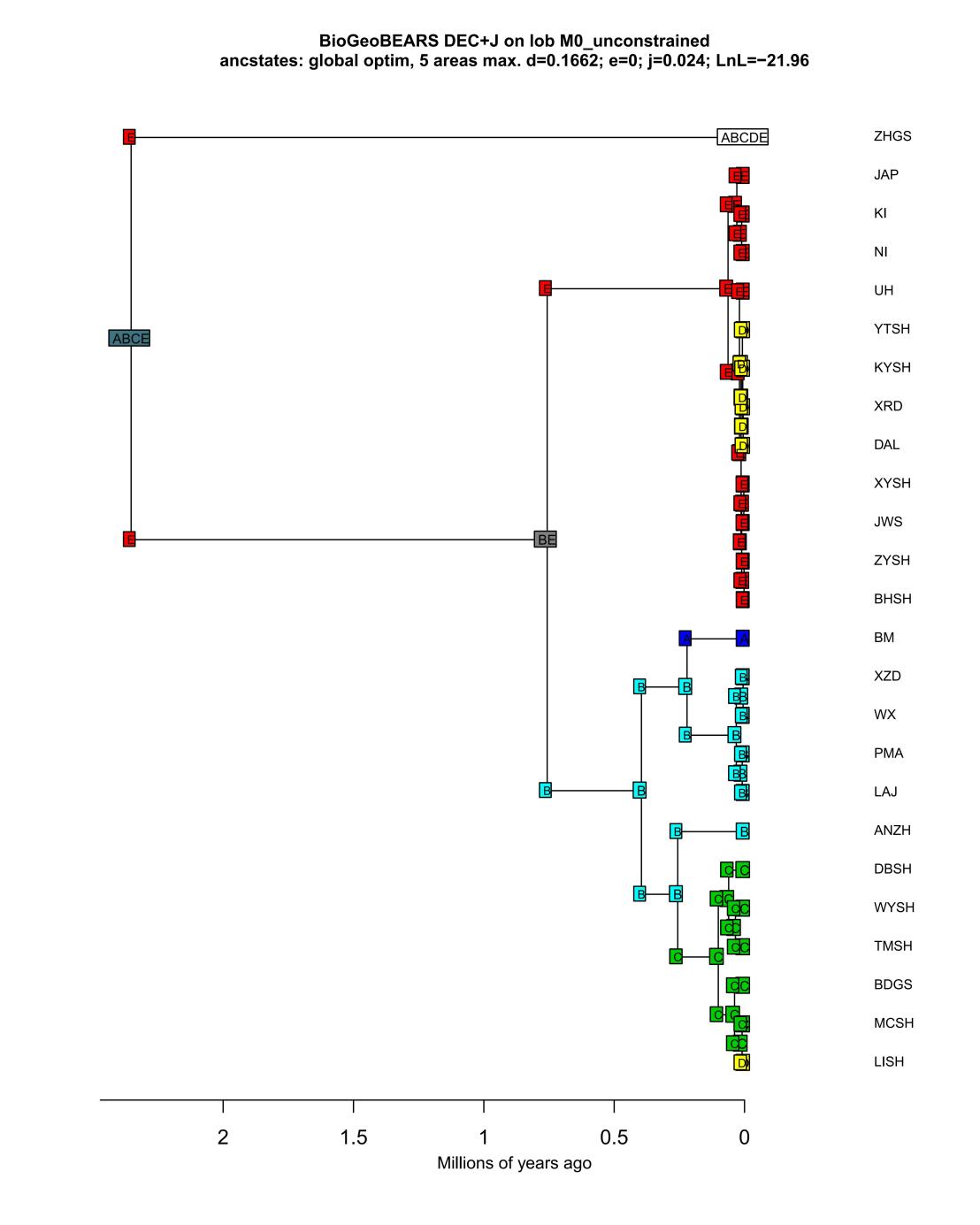
**

**Figure S6** Ancestral area reconstructions derived by DEC+j model usging BioGeoBEARS base on the BEAST-derived chronograms of 24 populations of *Lindera obtusiloba* with outgroup *L. erythrocarpa* (ZHGS) using 15 low-copy nuclear gene loci (the remaining 12 loci were not successfully sequenced in *L. erythrocarpa*) based on the estimated substitution rate, mean: 4.58 × 10-9 (95% highest-probability-density interval, HPD, : 1.66–12.69 × 10-9). Five geographic regions, Eastern Himalayan Province (A), Sikang–Yunnan Province (B), Central Chinese Province (C), North Chinese Province (D) and Japanese–Korean Province (E), were defined according to the floristic divisions [[1](#_ENREF_1)].
